# The International Practical Temperature Scale of 1968 in the Region 90.188 K to 903.89 K as Maintained at the National Bureau of Standards

**DOI:** 10.6028/jres.080A.053

**Published:** 1976-06-01

**Authors:** George T. Furukawa, John L. Riddle, William R. Bigge

**Affiliations:** Institute for Basic Standards, National Bureau of Standards, Washington, D.C. 20234

**Keywords:** Comparator, International Practical Temperature Scale of 1968, IPTS-68, oxygen point, platinum resistance thermometer, temperature standard, tin point, triple point of water, zinc point

## Abstract

The reproducibility of the International Practical Temperature Scale of 1968 (IPTS-68) in the region 90.188 K to 903.89 K as maintained at the National Bureau of Standards is discussed. The realizations of the triple point of water, the freezing points of zinc and tin, and the boiling point of oxygen are described. The average of the standard deviations of the resistance measurements at the triple point of water of 213 platinum resistance thermometers received for calibration over a two-year period corresponds to ±0.15 mK. The standard deviations of the resistance ratio *R*(*T*)/*R*(0°C) obtained with check thermometers employed for monitoring the zinc, tin, and oxygen point measurements correspond to ±0.28 mK, ±0.30 mK, and ±0.16 mK, respectively; the results of repeated calibrations with five thermometers show comparable reproducibility at the tin and oxygen points but the reproducibility is worse by a factor of two at the zinc point. When suitably packed for protection from possible mechanical shock platinum resistance thermometers can be shipped by common carrier and retain their calibrations.

## 1. Introduction

From 13.81 K (−259.34 °C) to 903.89 K (630.74 °C) the International Practical Temperature Scale of 1968 [1,2],^1^ referred to hereafter as IPTS-68, is based on nine defining fixed points, the platinum resistance thermometer (PRT) calibrated at these fixed points, and specified interpolation equations to relate the temperature and the resistance ratio,

W(T)=R(T)/R(0°C), (1)

where *R*(*T*) is the thermometer resistance at temperature *T* and *R*(0°C) is the resistance at 0°C. The defining fixed points are equilibrium states between phases of pure substances to which values of temperature have been assigned. (The isotopic composition is generally specified wherever it may vary sufficiently to have a significant effect on the equilibrium temperature.) The thermometer resistor must be annealed pure platinum, supported in a “strain-free” manner and have a value of *W*(100°C) not less than 1.39250. (Henceforth any reference to PRT indicates a thermometer of standards quality that meets the IPTS-68 specifications.) Below 0°C the *W* versus *T* relation of the PRT is given by

W(T)=W*(T)+ΔW(T), (2)

where W*(*T*) is the reference function defined by

$$T={\sum_{j=0}^{20}a_{j}\left[\frac{\ln\;W*\left(T\right)+3.28}{3.28}\right]}^{jK}$$ (3)

and Δ*W*(*T*) is a deviation function which is a polynomial in *T*[3]. (The coefficients a*_j_* of eq (3) are given in reference [2].) The range from 13.81 K to 273.15 K is divided into four subranges, each with its specified deviation function of the general form

$$\Delta W\left(T\right)=\sum_{i=0}^{n}k_{i}T^{i}$$ (4)

where *n* ⩽ 4. In the subrange 13.81 to 20.28 K, *n* = 3; 20.38 to 54.361 K, *n* = 3; 54.361 to 90.188K, *n* = 2; and 90.188 to 273.15 K, *n* = 4. The coefficients *k_i_* are determined by calibration at the fixed points and by the requirement that the first derivative, *d*Δ*W*(*T*)/*dT*, be continuous at the junction with the next higher subrange.

From 0 °C to 630.74 °C the values of temperature *t* are defined by

$$\begin{array}{l} t=t^{\prime }+0.045\left(\frac{t^{\prime }}{100{}^{\circ}C}\right)\left(\frac{t^{\prime }}{100{}^{\circ}C}-1\right)\\ \;\left(\frac{t^{\prime }}{419.58{}^{\circ}C}+1\right)\left(\frac{t^{\prime }}{630.74{}^{\circ}C}+1\right){}^{\circ}C, \end{array}$$ (5)

where *t*′ is defined by

$$W\left(t^{\prime }\right)=R\left(t^{\prime }\right)/R\left(0{}^{\circ}C\right)=1+At^{\prime }+B{t^{\prime }}^{2}.$$ (6)

By definition *t* and *T* are related by *t = T* − 273.15 K[2]. Equation (6) is equivalent to

$$t^{\prime }=\left\{\frac{1}{\alpha }[W(t^{\prime })-1]+\delta \left(\frac{t^{\prime }}{100{}^{\circ}C}\right)\left(\frac{t^{\prime }}{100{}^{\circ}C}+1\right)\right\}{}^{\circ}C,$$ (7)

where

α=A+B×100°C (8)

and

$$\delta =-\frac{B{\left(100{}^{\circ}C\right)}^{2}}{A+B\times 100{}^{\circ}C}.$$ (9)

The thermometer constants *R*(0°C), *A*, and *B* are determined from resistance measurements at the triple point of water, the steam point or the tin point, and the zinc point. The constants *α* and δ are derived from the constants *A* and *B* according to eqs (8) and (9), respectively.

The accuracy of thermometry employing a well calibrated PRT depends primarily upon precise measurements of relative resistances to determine *W(T*) accurately. In the calibration of PRT’s, there are required, in addition, accurate realizations of the defining fixed points to obtain the thermometer constants. At the National Bureau of Standards (NBS) the defining fixed points: the triple point of water, the tin point, and the zinc point are maintained and are employed regularly in the calibration of PRT’s. The defining fixed points below 0 °C are “maintained” at present by reference standard PRT’s of the capsule type. In an earlier paper, the NBS–IPTS-68 scale between 13.81 and 90.188 K maintained by the reference standard PRT’s was described and the reproducibility of the calibrations, in terms of the NBS–IPTS-68 scale, of capsule type PRT’s received for calibration during a 2½-year period was shown [4]. The present paper deals with the calibration of long-stem type PRT’s (and some capsule type PRT’s in special holders) in the range 90.188 to 903.89 K. The fixed points that are maintained, the method of calibration, and the reproducibility of the results are discussed.

## 2. Fixed Points

### 2.1 Triple Point of Water

Of the thirteen defining fixed points^2^ that are specified in the text of the IPTS-68 the triple point of water, henceforth referred to as TP, is the most important. (The Thermodynamic Kelvin Temperature Scale is defined by assigning 273.16 K to the temperature of the *TP* and the thermodynamic unit of temperature, kelvin, is defined as 1/273.16 of the thermodynamic temperature of the *TP* [2].) The PRT temperature scale as defined by the IPTS-68 utilizes the resistance ratio, *R(T*)/*R*(0°C); consequently, the accuracy of every temperature measurement depends on the accuracy of two observations, *R(T*) and *R*(0°C). Above 0°C, any errors in *R*(0°C) are amplified in the resistance ratio *R(T*)/*R*(0°C). (Hereafter, the resistance ratio *R(T*)/*R*(0°C) will be referred to as *W*(*T*).) In practice, the *R*(0°C) of the PRT is determined from measurements at the TP; hence, the accuracy of *R*(0°C) of the PRT ultimately depends on the accuracy of the measurements at the temperature of the TP and on the reproducibility of the TP. In a recent intercomparison of the TP temperatures of a bank of fifteen TP cells at the NBS, six observations were made on each cell over a period of three days; the averages of temperatures observed on each cell were within ±0.1 mK [5].

**Figure 1:**
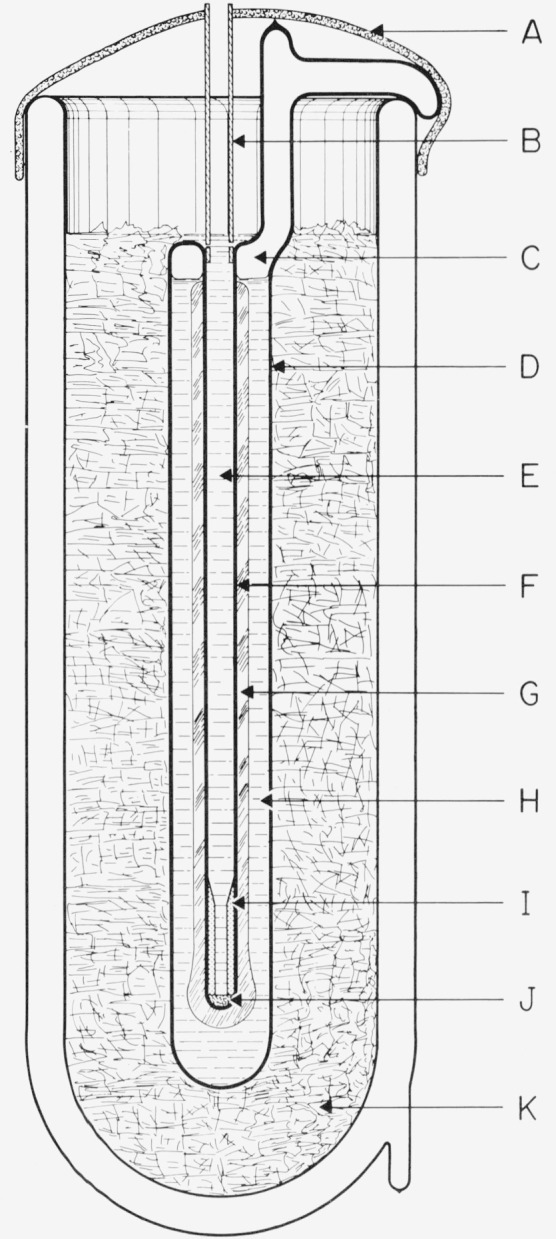
Water triple point cell in an ice bath. Heavy black felt for shielding against ambient radiation.Polyethylene tube for guiding the SPRT into the thermometer well.Water vapor.Borosilicate glass cell.Water from ice bath.Thermometer well (precision bore).Ice mantle.Air-free water.Aluminum bushing with internal taper at upper end to guide the SPRT into the close-fitting inner bore.Polyurethane sponge.Finely divided ice and water.

Figure 1 shows the design of the TP cell employed at the NBS. The sealed borosilicate glass cell contains only ice, water, and water vapor. The thermometer well is constructed of precision bore tubing. The extension at the top serves as a handle or as a support, as shown in the figure. By suitably positioning the cell most of the “gas” can be trapped in the extension and the amount of air can be estimated from the size of the bubble, if any, that remains. (At room temperature when the TP cell is positioned with the bottom end up, the bubble trapped in the extension is under a hydrostatic head of water that corresponds to a pressure slightly higher than the vapor pressure of water. Hence, if the bubble contains only water vapor, it should collapse when the TP cell is turned bottom end up.)

To prepare the TP cell for use, the cell is first cooled by immersing it in a bath of shaved ice for about one-half hour or so. The cell is then mounted in the ice bath with the opening of the reentrant thermometer well above the ice-water level and the thermometer well is wiped dry, e.g., by using absorbent paper on the end of a rod or a tube. A mantle of ice is frozen around and immediately next to the thermometer well by filling the thermometer well with crushed Dry Ice. The well is maintained full of Dry Ice for about 20 min, after which all of the Dry Ice is allowed to evaporate. (It is important to keep the well completely full of Dry Ice during the 20-min period. If the Dry Ice level in the well is allowed to fall several inches and then the well is refilled, the ice mantle is apt to crack. Although the crack often “heals,” the desired triple-point temperature of the “inner melt” may not be achieved if a crack in the ice mantle later extends from the well surface into the surrounding liquid water of the cell. The impurities, if any, in the water are expected to become concentrated in the remaining liquid water that surrounds the ice mantle of the cell. This less pure liquid water should not be allowed to mix with the inner melt near the level of the thermometer resistor.)^3^ Usually dendritic ice crystals form around the bottom of the well on the introduction of the first few pieces of Dry Ice. As more Dry Ice is introduced to fill the thermometer well, a clear coating of ice forms around the well and gradually grows to about 7-mm thickness during the 20 min the well is maintained full of Dry Ice. About 1-mm additional thickness of ice is formed when the remaining Dry Ice, after the 20-min period, is allowed to evaporate. Also, the ice mantle around the bottom end of the well, where the thermometer coil is normally located, becomes somewhat thicker (see fig. 1). After the Dry Ice is completely gone the cell is lowered deeper into the ice bath and the thermometer well is allowed to fill with ice water.

During the 20 min when the thermometer well is maintained full of Dry Ice, there is a strong tendency for the water to freeze solidly across at the top surface of the water in the cell. If this layer of ice forms a strong bond with the thermometer well and the outer cell, subsequent freezing of the water below this layer can develop enough pressure to possibly rupture the glass cell.^4^ Therefore, whenever a layer of ice is about to bridge (or has bridged) between the thermometer well and the outer cell wall, the outer cell wall near the upper water surface should be warmed to melt the surface ice. This may be accomplished by raising the cell out of the ice bath briefly and warming the cell near the unwanted ice with the hands while gently shaking the cell sideways to wash and, therefore, melt the ice with the warmer water of the cell. During this time the Dry Ice level in the thermometer well should not be allowed to fall too low.

If the cell is raised high enough to see the mantle during the freezing process, the magnification by the cylinder of water will give the appearance that the ice mantle is thick enough to contact the outer wall although the mantle may actually be much thinner (1 or 2 mm thick).^5^ During the freezing of the mantle, if the cell has enough vapor space, the cell may be momentarily inverted, while keeping the Dry Ice from falling out of the thermometer well, to see the mantle without the effect of magnification and, therefore, see the true thickness of the mantle. The cell should be inverted only when the thermometer well is cold enough so that the ice mantle would remain fast to the wall and, therefore, would not float upward against the “bottom” of the cell. If the cell is inverted after the inner melt is made the ice mantle will float upward to the bottom of the cell and the chemical composition of the inner liquid would be expected to be altered by the outer liquid.^6^

The temperature of the water-ice interface of an inner melt is the fixed-point temperature of the TP cell. The inner melt is formed by inserting a solid glass rod at ambient temperature into the thermometer well of the TP cell filled with ice-bath water; thereby, a thin layer of ice adjacent to the thermometer well is melted. The existence of this inner melt is tested by giving the cell a sharp rotatory impulse about the axis of the well and observing if the ice mantle spins or moves freely around the thermometer well. The TP cell usually equilibrates to essentially constant temperature within 30 min to 1 h. after preparation using Dry Ice. In most applications at the NBS the TP cell is prepared at least one day prior to use.

During storage of the TP cell in the ice bath, the frozen mantle of ice grows slowly and irregularly until it contacts and attaches itself to the outer walls of the cell. The cell is occasionally removed from the ice bath and the excess ice melted by warming with the hands or with water. (As mentioned earlier the extension on the type of TP cells used at the NBS serves as an indicator of the amount of water frozen in the cell. When the water level reaches the vertical extension, the amount of ice formed is usually considered excessive.) The growth of ice in the TP cell while stored in the ice bath can be reduced by insulating the TP cell with a sheet of plastic foam or by placing the TP cell in a Dewar flask which is in turn immersed in the ice bath. By means of a suitable insulation and occasional melting of excess ice the same freeze of the TP cell may be used in an ice bath for a number of months withoug freezing a new mantle. Moreover, in the PRT calibration laboratory of the NBS the ice bath for the TP cells is installed inside a small freezer chest to reduce the melting of the ice and, thereby, reduce the frequency of replenishing of the ice in the ice bath.

The TP cell is employed with the PRT as follows. Referring to figure 1, a small soft plastic foam (J) is first placed at the bottom of the thermometer well to reduce the mechanical shock when the PRT is inserted. A closely fitting aluminum bushing (I) about 5 cm long is placed above the foam to reduce the external self heating of the PRT (see sec. 4 on self heating). The bushing is constructed of aluminum instead of the heavier copper so that the bushing would gently sink to the bottom on top of the foam when it is inserted into the thermometer well full of water. The bushing has a tapered hole at the top to gently center the PRT when it is inserted into the thermometer well. To avoid upward displacement of the bushing by a “pumping” action when the PRT is inserted, the bushing is not too close fitting with the PRT sheath. To eliminate the effect of the room radiation (particularly the ceiling lights) a heavy black felt cloth (A) is placed over the top of the cell and ice bath except for a hole through which the PRT is inserted.^7^ The PRT is precooled in the ice bath that surrounds the TP cell before it is inserted into the cell. A polyethylene plastic tube (B) helps to guide the PRT gently into the thermometer well. Before the final measurements on the PRT are made the thermometer current is on continuously for at least 5 min (with the Mueller bridge nearly balanced) for the thermometer to come to temperature equilibrium.

The temperature of the water-ice interface of the inner melt where the PRT resistor is immersed (more precisely, the location of the mid-point of the resistor) is slightly lower than the temperature of the TP. The temperature depression is 7×10^−6^ K per cm of water column [2]. The depth of immersion of the PRT is taken to be the distance between the upper water surface of the TP cell to the mid-point of the PRT resistor. The TP cells in use at the NBS have well depths of 32 cm or 36 cm, the TP cells with 32 cm wells being the most common and most recent. The depth of immersion of the PRT is about 29 cm or 33 cm, which corresponds to the temperature depression of the TP of 200 *μ*K or 230 *μ*K, respectively; therefore, the temperature of the TP cell at the mid-point of the PRT resistor is either 0.00980 °C or 0.00977 °C, depending upon the immersion depth of the TP cell being used.

The value of *R*(0°C) is obtained by converting the observed values of *R*(TP) employing equation (6) (see sec. 1, Introduction). Because of the small value of *t*′ at the TP (*t* = 0.00980°C ≅ *t*′ or *t* = 0.00977 °C ≅ *t*′), the term containing *t*′^2^ is negligible and the average value of *A* (3.98485×10^−3^ °C^−1^), found for PRT’s calibrated at the NBS in the past years, is employed in the conversion. The uncertainty in the adjustments of the value from *R* (TP) to *R*(0°C) in this manner is less than ± 1 × 10^−8^ *R*(0°C) for PRT’s that meet the IPTS-68 specification that *W*(100°C) be not less than 1.39250. Hence, in obtaining *R*(0°C) from *R*(TP), the uncertainty contributed by this procedure is negligible.

### 2.2. Zinc Point

In place of the normal boiling point (NBP) of sulfur or the sulfur point the equilibrium state between the solid and liquid phases of zinc at 1 standard atmosphere (henceforth referred to as the freezing point of zinc or the zinc point) was recommended as a defining fixed point with the value of 419.505 °C on the IPTS–48 (Revised 1960) [7, 8]. The presently used IPTS-68 assigns the value 419.58°C to the freezing point of zinc. Prior to 1966 the sulfur point was maintained at the NBS for the calibration of PRT’s; however, since 1966 the zinc point has been maintained instead of the sulfur point.^8^

#### 2.2.1. Zinc-Point Cell

**Figure 2:**
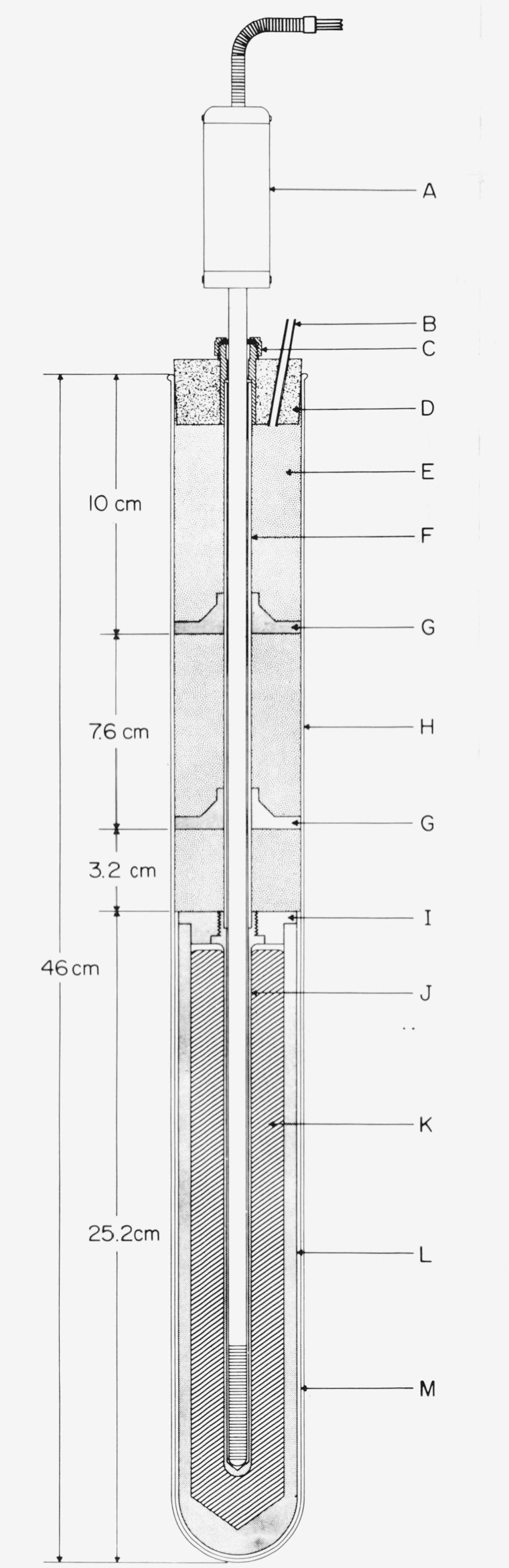
Metal freezing-point cell. Platinum resistance thermometer.To helium gas supply and pressure gauge.Thermometer stem seal with silicone rubber gasket.Silicone rubber stopper.Insulation, washed Fiberfrax.Thermometer guide tube, borosilicate glass.Heat shunt, graphite.Borosilicate glass cell.Graphite cap (lid).Graphite thermometer well.Metal sample, zinc or tin.Graphite crucible.Insulation, Fiberfrax paper.

The freezing-point cell employed to realize the zinc point is illustrated in figure 2.^9^ A bank of such cells assembled from zinc samples (SRM–740) of greater than 99.9999 percent purity is available for the calibration work. The freezing points of these zinc-point cells agree within ±0.1mK. The residual resistivity ratios (the ratio of the electrical resistance at 273 K to that at 4 K) of specimens taken from zinc bars from which the SRM 740 zinc samples were prepared ranged from 33,000 to 38,000 [9], indicating that the zinc samples have very high purity.

The zinc samples had been prepared from a starting material that was selected from a lot of electrolytic zinc of 99.99 + percent purity. The material was vacuum distilled twice and zone refined with 20 passes. After the leading and trailing ends of the zone refined bars were cropped, they were homogenized and cast in fused quartz boats in the form of semicylindrical bars about 5 cm across the flat side. The bars (approximately 61 cm long) were delivered to the National Bureau of Standards – Office of Standard Reference Materials (NBS–OSRM) individually sealed in argon-filled polyethylene bags. The zinc samples (1250 g) for each of the freezing-point cells were received from the NBS–OSRM in the form of two half cylinders (each about 8 cm long) sealed in a polyethylene bag. The samples had been cut from the bars with a carbide tipped tool; they were then etched in high-purity dilute nitric acid, rinsed with distilled water, and air dried.

Because of the relatively high vapor pressure of zinc at its melting point {about 13 Pa (0.1 Torr) [10]^10^}, the zinc samples were melted into the high-purity graphite crucibles by induction heating under purified argon atmosphere. The graphite thermometer well was then inserted and the cell was assembled in the form shown in figure 2. Details of the assembly procedure are the same as those described for tin in references [11, 12].

#### 2.2.2. Furnace for the Zinc-Point Cell

**Figure 3:**
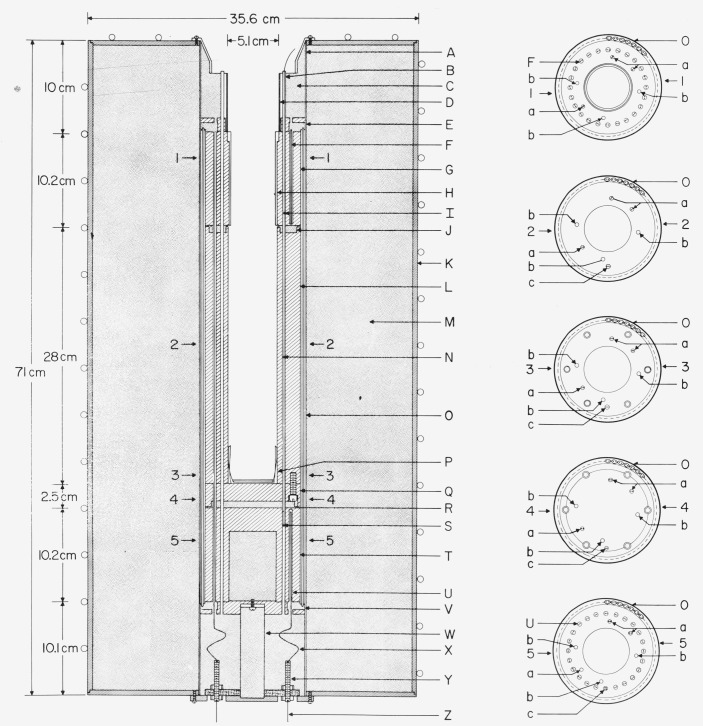
Schematic of furnace body and core. Stainless steel tube, 11.4 cm o.d. × 0.76 mm wall.Stainless steel tubes (6): 3.20 mm o.d. × 0.013 mm wall.Insulation, Fiberfrax mats.Stainless steel tube.Insulation, Fiberfrax mats and mica sheets.Control heaters for top core block.Top core block.Sleeve (aluminum or Inconel) for metal freezing point cell.Elevation of thermocouple junction (Chromel-P/Alumel), top core block. (See a.)Insulation, Fiberfrax sheets.Brass shell, 4.8 mm thick.Center core block.Insulation, bulk Fiberfrax.Elevation of thermocouple junction (Chromel-P/Alumel), center core block. (See a.)Main heaters, held on with Inconel “garters.”“Spider” for centering the freezing point cell.Center core block end plate.Insulation, Fiberfrax sheets.Elevation of thermocouple junction (Chromel-P/Alumel) bottom core block. (See a.)Bottom core block.Control heaters for bottom core block.Insulation, Fiberfrax mats and mica sheets.Stainless steel tube support, 2.5 cm o.d. × 0.51 mm wall × 11.4 cm long.Heater leads.Posts for heater leads.To electric power.
Wells (B) for control thermocouples (See I, N, and S.)Wells (B) for testing the temperature profile of furnace core.Leads from top core block heaters.

The design of the furnace employed with zinc-point cell is shown in figure 3. The furnace core consists of cylindrical blocks of aluminum [top (G), center or main (L), and bottom (T)]. The space surrounding the core is packed with Fiberfrax insulation. The temperature of the center core (L) is controlled by means of an absolute thermocouple and the main heater (O). The temperatures of the top (G) and bottom (T) blocks are controlled relative to the center block temperature by means of differential thermocouples and heaters [(F) and (U), respectively] in the blocks. The electric power to the heaters is controlled automatically from the indication of the corresponding thermocouples.

#### 2.2.3 Preparation of Zinc-Point Freeze

Liquid zinc is found to supercool only 0.02 to 0.06 °C. On the day prior to the zinc-point calibration of the PRT’s the furnace control is set at a temperature about 5 °C above the zinc point and the zinc sample is melted overnight. The following morning after inserting a monitoring PRT in the cell, the furnace control is reset to a temperature about 4 °C below the zinc point. After the PRT indicates that recalescence has occurred and about 10 min have passed, the monitoring PRT is removed and two borosilicate glass rods are inserted successively into the thermometer well for about 3 min each to induce an inner freeze immediately next to the thermometer well. Also, the furnace control is reset to a temperature 1 °C below the zinc point. After withdrawing the second borosilicate glass rod, the cold monitoring PRT is inserted into the thermometer well. By following this procedure freezes of 12 to 14 h. durations are obtained. With cells assembled using SRM–740 zinc standard the change observed in the freezing point while the first 50 percent is frozen is less than 0.2 mK.

The resistance of the monitoring or check PRT is determined approximately 45 min after inserting it cold into the zinc-point cell. (Meanwhile the test PRT is being preheated in an auxiliary furnace held about 20 °C above the zinc point.) After completion of the measurements on the check PRT it is removed from the zinc-point cell and the test PRT is withdrawn from the preheating furnace and quickly inserted into the zinc-point cell so that the thermometer temperature during the insertion would be slightly below that of the cell. (The PRT is inserted slightly colder than the cell temperature to avoid melting and loosening the solid zinc mantle around the thermometer well.) When the PRT is preheated to a temperature close to that of the cell temperature it reaches temperature equilibrium within a few minutes (see fig. 35, Monograph 126 [12]). The resistance readings of the test PRT are started about 15 min after inserting it into the zinc-point cell. (Meanwhile, the second test PRT is being preheated in the auxiliary furnace.) After completion of the measurements on the first test PRT, it is replaced in the zinc-point cell by the second test PRT. A maximum of six test PRT’s are calibrated in any single zinc freeze. After the last test PRT is calibrated it is replaced with the preheated check PRT. This second reading with the check PRT must not differ from the first reading by more than 0.5 mK; if the difference is larger, the measurements on some of the test PRT’s that were calibrated last will be repeated using another zinc freeze. The test PRT’s on which the measurements are to be repeated are decided on the basis of the elapsed time since the start of the freeze and the change in the freezing temperature that is observed with the check PRT.

Because of the effect of the hydrostatic head of liquid zinc, the temperature at the height where the mid-point of the PRT sensor is located in the zinc-point cell is slightly hotter than the equilibrium freezing temperature of zinc at 1 atm pressure as defined in the IPTS-68. (The effect of the hydrostatic head is 27 *μ*K per cm of liquid zinc [2].) For the NBS zinc-point cell the height of the liquid zinc column above the mid-point of the sensor is 18 cm; therefore, the temperature at the height of the mid-point of the PRT sensor is 419.5805 °C.

### 2.3. Tin Point

The equilibrium state between the solid and liquid phases of tin at 1 standard atmosphere (231.9681 °C, henceforth referred to as the freezing point of tin or the tin point) is an alternative to the normal boiling point of water (steam point) as a defining fixed point on the IPTS-68. Prior to 1966 the steam point was maintained at the NBS for the calibration of PRT’s; however, since 1966 the tin point has been maintained instead of the steam point.

#### 2.3.1. Tin-Point Cell

The freezing-point cell employed to realize the tin point is designed similar to the zinc-point cell (see fig. 2). A bank of tin-point cells assembled from tin samples (SRM–741) of nominally 99.9999 percent purity is available for the calibration work. The freezing points of these cells agree within ±0.1 mK [11]. The residual resistivity ratios of specimens taken from tin bars from which SRM–741 tin samples were prepared varied from 28,000 to 43,000 [13], indicating that the tin samples have very high purity.

The tin samples had been prepared by initially electrolyzing commercially refined tin and zone refining the electrolyzed product by at least 20 zone passes. The purified samples were homogenized and cast into semicylindrical bars about 5 cm across the flat side and about 60 cm long. Each of the tin samples (1300 g each) were received from the NBS–OSRM in the form of two half cylinders (each about 10 cm long) sealed in a polyethylene bag. The samples had been cut from the bars with a carbide tipped tool. To remove surface contamination they were etched first in 40 percent hydrochloric acid solution and then in a solution of 40 percent hydrochloric acid plus 10 percent nitric acid; they were washed in distilled water and in ethyl alcohol and air dried.

The tin samples were melted into the high-purity graphite crucible by induction heating under high vacuum. (The vapor pressure of tin at its melting point is estimated to be about 6×10^−21^ Pa [10].) The graphite thermometer well was then inserted and the cell was assembled in the form similar to that of the zinc cell shown in figure 2. Details of the assembly procedure are given in references [11, 12].

#### 2.3.2. Furnace for the Tin-Point Cell

The design of the furnace employed with the tin-point cell is the same as that used with the zinc-point cell (see fig. 3). Initially the sleeve (H) for enhancing the thermal contact between the heat shunts (G, fig. 2) of the tin-point cell and the furnace was aluminum; however, in recent measurements an Inconel sleeve, similar to that used in the zinc-point furnace, has been used in the tin-point furnace.

#### 2.3.3. Preparation of the Tin-Point Freeze

Liquid tin has been found to supercool as much as 25 °C, depending upon the temperature and length of time the metal was allowed to remain melted. Impurities (e.g., Fe) seem to reduce the degree of supercool [14]. If the freeze were initiated with the tin-point cell in the furnace, the furnace block temperature could not then be raised fast enough to avoid excessive freezing of tin. Therefore, the tin freeze is initiated by employing the “outside nucleated freeze” technique described by McLaren [15]. The tin sample is melted overnight in the furnace held at a temperature about 3°C above the freezing point. In the morning after inserting a monitoring PRT in the thermometer well, the furnace control is reset down to a temperature 1°C below the tin point. (A different monitoring PRT is used with the tin-point cell from that used wtih the zinc-point cell, so that any possible effect on the PRT when used at another fixed point would be avoided. As with the zinc-point cell the monitoring PRT used with the tin-point cell serves also as the check PRT.) When the monitoring PRT indicates that the tin sample has cooled to near the freezing point, the cell is withdrawn from the furnace and held out in room temperature. When the PRT indicates that the sample has started to recalesce, the cell is quickly reinserted into the furnace. Following this procedure a freeze duration of 12 to 14 h is obtained. With the tin-point cells containing SRM–741 tin standard, after about 5 percent of the initial “freeze,” the temperature change in the following 50 percent of the freeze is less than 0.1 mK. The resistance of the monitoring or the check PRT is determined approximately 45 min after reinserting the tin-point cell into the furnace. (Meanwhile a test PRT is being preheated in an auxiliary furnace held 20 °C above the tin point.) After completion of the measurements on the check PRT it is removed from the tin-point cell and the test PRT is withdrawn from the preheating furnace and quickly inserted into the tin-point cell so that the thermometer temperature during the insertion is slightly above that of the cell. In actual practice it is not certain whether any melting or any additional freezing occurs upon insertion of the PRT. In either event the preheated PRT reaches temperature equilibrium within a few minutes (see fig. 31 Monograph 126 [12]). The resistance readings of the test PRT are started 15 min after inserting it into the tin-point cell. (Meanwhile the second test PRT is being preheated in the auxiliary furnace.) After completion of the measurements on the first test PRT it is replaced in the tin-point cell by the second test PRT. As with the zinc-point cell a maximum of six test PRT’s are calibrated in any single tin freeze. After the last test PRT is calibrated it is replaced with the check PRT. The requirement of the second reading of the check PRT is that it shall not differ from the first reading by more than 0.5 mK; if the difference is larger, the measurements on some of the test PRT’s that were calibrated last will be repeated using another tin freeze. The test PRT’s on which the measurements are to be repeated are decided on the basis of the elapsed time since the start of the freeze and the change in the freezing temperature that is observed with the check PRT.

Because of the effect of the hydrostatic head of liquid tin, the temperature where the midpoint of the PRT sensor is located during measurements in the tin-point cell is slightly higher than the equilibrium freezing temperature of tin at 1 atm pressure as defined in the IPTS-68. (The effect of the hydrostatic head is 22 *μ*K per cm of liquid tin [2].) For the NBS tin-point cell the height of liquid tin column above the midpoint of the PRT sensor is 18 cm; therefore, the temperature at the midpoint of the PRT sensor is 231.9685 °C.

### 2.4 Normal Boiling Point of Oxygen

**Figure 4:**
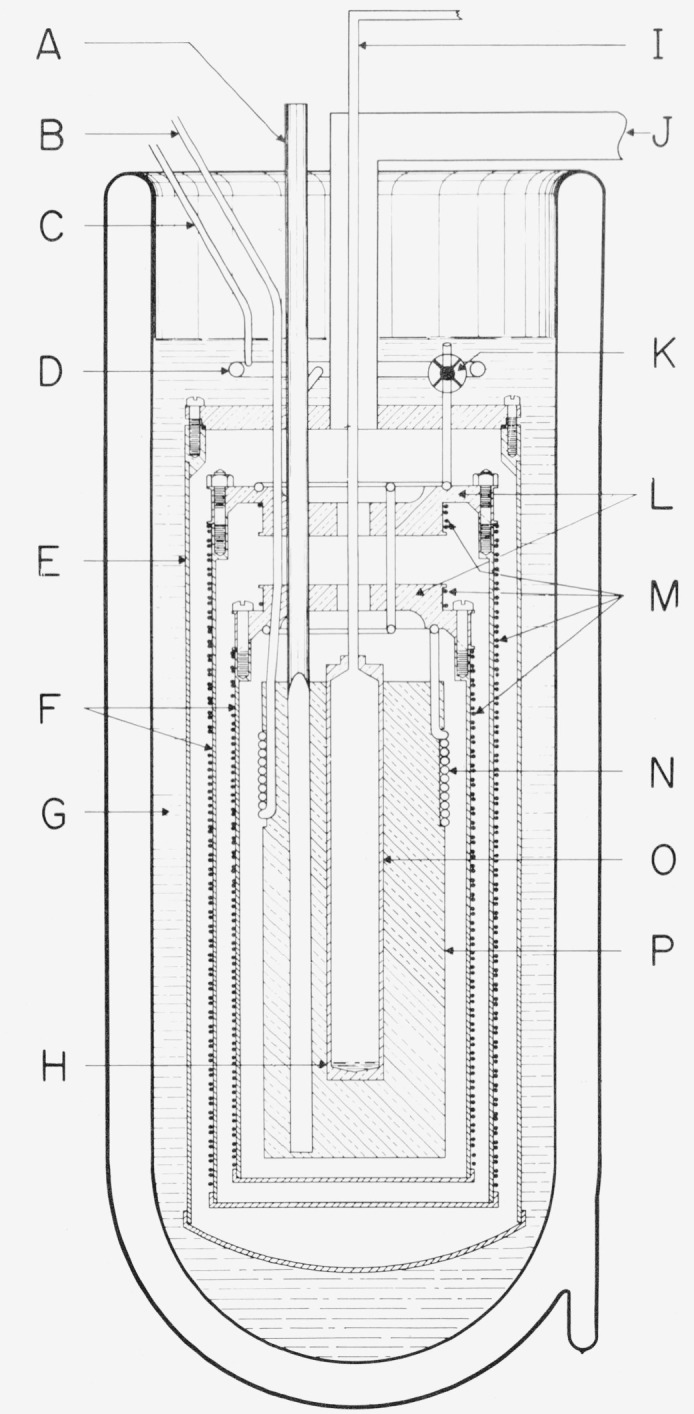
Cryostat for comparison calibration of thermometers with reference standards at the oxygen normal boiling point. Thermometer well; eight wells are located around the copper block. The SPRT’s are sealed at the top with molded silicone rubber.Tube to vacuum pump to draw liquid nitrogen through cooling tubes.Tube to helium gas supply.Manifold for distributing helium gas to the thermometer wells.Envelope (brass).Radiation shields (copper).Liquid nitrogen.Liquid oxygen. (Not employed in comparison calibrations.)Vapor-pressure tube, to differential pressure diaphragm and manometer.High vacuum line.Valve to control the liquid nitrogen input for cooling.Top heat shields, to control thermometer well and thermometer stem temperatures.Heaters.Cooling tubes (thin-wall Monel).Oxygen bulb.Copper block.

The calibration of the PRT’s at the normal boiling point of oxygen (−182.962, oxygen point) is realized by reference to the NBS–1955 temperature scale adjusted to correspond to the IPTS-68 (see reference [4] for the discussion of this NBS–IPTS-68 scale between 13.81 and 90.188 K maintained on capsule-type reference standard PRT’s). The temperature (hotness) assigned to the oxygen point is also maintained by long-stem type reference standard PRT’s which are intercompared with the capsule type PRT reference standards. Calibrations near the oxygen point are obtained by intercomparing the test PRT’s with a long-stem type reference standard PRT in the apparatus shown in figure 4. Eight thin-walled Monel tubes (A) extend into the copper block (P); five tubes are 7.9 mm o.d. to accommodate regular long-stem type PRT’s, one is 10.3 mm o.d. for 9 mm PRT’s, and two tubes are 12.7 mm o.d. to accommodate capsule type PRT’s in holders. The tubes are soldered to the copper block as well as to the copper flanges at the various stages of the apparatus shown in figure 4. The thermometer stems are sealed at the top of the wells by a molded band of soft silicone rubber. The thermometer wells are filled with helium gas to a pressure that is slightly above atmospheric (see helium gas distributing manifold (D) in fig. 4). The helium gas enhances the thermal contact between the thermometer and the wall of the well and reduces the chance of condensible gases entering the wells.

The apparatus is prepared for calibration by evacuating through (J) and immersing in liquid nitrogen to the level shown in figure 4. The copper block (P) and shields (F and L) are cooled by admitting nitrogen gas and/or liquid nitrogen through valve (K) just below the liquid nitrogen surface. The cool nitrogen vapor flows through coils (N) attached to the two top shields and to the copper block and exits through (B) to a large-capacity vacuum pump. When the temperature of the copper block (P) is cooled nearly to the oxygen point, the valve (K) is closed and the nitrogen in the cooling coils is removed by pumping. The temperature of the copper block is adjusted to within 1 K of the oxygen point [either by heating or, if necessary, by opening valve (K)] and the two shields are controlled at the temperature of the block. (Calibrations of the PRT’s are performed within 1 K of the oxygen point.) When the temperatures of the copper block and the two shields are nearly the same, the inner shield is allowed to “float” without electric power input. The outer shield is controlled relative to the temperature of the copper block by means of differential thermo-couples and shield heaters. The floating shield tends to dampen the effects of temperature gradients and short term variations in the control of the outer shield. The electric power in the shield heaters is controlled automatically from the emf output of the thermocouples. The PRT’s are tempered where the tubes pass through liquid nitrogen and where the two shields are soldered to the tubes (A) before entering the copper block (P).

In the calibration of the PRT’s the resistance measurements are carried out on the test PRT and the reference standard PRT simultaneously using two Mueller bridges. Measurements are made as rapidly as possible with the commutating switching order NRRNNRRN of the Mueller bridges. As a part of the calibration process the reference standard PRT is checked by comparing with a second reference standard PRT. The temperature at which the resistance of the test thermometer is observed is calculated from the observed resistance of the reference standard PRT and its *R* versus *T* relation. (Actually, the analysis is performed in terms of the *W* versus *T* relation.)

## 3. Resistance Measurements

The resistance measurements are performed with Mueller bridges. The bridge is basically an equal- arm Wheatstone bridge with modifications to permit commutation of the four PRT leads so that the lead resistances cancel when two bridge readings are averaged. Other modifications include a provision for adjusting the ratio arms to equality and a provision for commutating the ratio arm connections at the same time the PRT leads are commutated, so that small deviations from unity of the ratio arms would be negligible. (For details of the bridge design see reference [12].) A galvanometer photocell amplifier plus a secondary “display” galvanometer are used for balance detection. Under favorable circuit conditions the unbalance can be detected within a few nanovolts. In the process of determining the resistance of the PRT the bridge current is reversed to eliminate the effects of any spurious emf’s. The current reversal method also doubles the apparent null detector sensitivity. The observations are made to the nearest 10 *μ*Ω in the following sequence of commutator switch positions: NRRN. (At the oxygen point, observations in this sequence are obtained twice.)

The Mueller bridges are calibrated once a year. (Previously the bridges were calibrated twice a year.) First the bridge is internally calibrated for linearity in terms of the resistance X(10) of the 1-Ω dial switch. The resistance of a standard resistor (close to 10 Ω), which had been calibrated in terms of the absolute ohm maintained at the NBS, is then measured using the bridge^11^. These data are used to obtain corrections to the nominal values of the switch positions in terms of the absolute ohm. Bridge calibrations are obtained to 10^−7^ Ω for the 1-ohm dial switch and for the lower dial switches and to 10^−6^ Ω for 10 and 100-ohm dial switches. For details of the Mueller bridge calibration see the appendix of reference [12].

A small residual unbalance exists in the Mueller bridge even with all measuring switches set on zero because of the resistances of the leads that connect the resistor network and the resistances of the switch contacts. The Waidner-Wolff elements [16] contribute also to the residual resistance. In the bridge design a resistor (about 0.98 Ω) is placed in series with the 0.1-ohm decade resistors (which operates “subtractively,” see reference [12]) to balance the remainder of the residual resistance of the Waidner-Wolff elements in the adjacent arm and the necessary copper connecting wires in the bridge when all switches are set to zero. The deviation from balance is the “bridge zero.” The bridge zero is subtracted algebraically from the dial readings of all measurements. Because of the changes in the switch contact resistance and changes in the temperature of some bridge components the bridge zero varies during the day. Each day the bridge is used the wiping contacts of the switches are exercised, the mercury contacts are cleaned and the mercury replaced, and the bridge zero is read and estimated to 10^−7^ Ω. During the day two or three bridge zero measurements are obtained.

## 4. Self-Heating Effects

The measurement of the PRT resistance involves passing an electric current through the resistor. The Joule heating (or *i*^2^*R* heating) raises the temperature of the resistor, its support, and its leads above that of the surrounding until a steady state condition is reached where the Joule heating equals the rate heat is dissipated to the surrounding medium. (If the temperature of the surrounding medium remains or becomes constant, e.g., as in a fixed-point cell, a steady PRT resistance reading can be attained. Otherwise, the PRT resistance will continue to change depending upon the Joule heating in the PRT resistor and upon other sources of energy exchange of the system.) The time required to reach the steady state condition depends upon the thermal diffusivities of the thermometer components and of the surroundings and upon the thermal resistances between them. In some situations as much as 5 min or longer may be required to reach the steady state condition. The magnitude of the self heating depends upon the Joule heating and upon the rate of heat transfer from the PRT resistor. At a given temperature the heat conduction from the thermometer resistor to the outer surface of the protective sheath remains essentially constant since the geometry and the thermal conductivity of the thermometer components usually remain unchanged with use. However, the heat conduction from the outer surface of the thermometer sheath to the surrounding medium depends upon the degree of thermal contact of the thermometer with the “surrounding heat sink.” Therefore, the heat conduction external to the thermometer depends upon the conditions of use. The self heating that is intrinsic to the thermometer is referred to as the *internal* self heating of the PRT. The self heating that is associated with the heat conduction external to the thermometer is referred to as the *external* self heating. The combined self heating is referred to as the *total* self heating. The total self heating may be expressed as a resistance change or as a temperature change corresponding to the resistance change and, strictly speaking, at a given temperature and specified surroundings.

At low electric power levels the total self heating is very nearly linear with respect to the power that is generated in the PRT resistor. At the NBS, measuring currents of 1 and 2 mA are usually used with PRT’s of *R*(0 °C) of about 25 Ω to determine the total self heating. If *R*_1_ is the resistance reading at a current of *i*_1_ and *R_2_* is the reading at a current of *i*_2_ the resistance *R*_0_ at zero current is given by

$$R_{0}=R_{1}-i_{1}^{2}\left(R_{2}-R_{1}\right)/\left(i_{2}^{2}-i_{1}^{2}\right).$$ (10)

For *i*_1_ and *i_2_* of 1 and 2 mA, respectively,

$$R_{0}=R_{1}-\left(R_{2}-R_{1}\right)/3.$$ (11)

Thus, at 1 mA thermometer current the total self heating is (*R_2_* − *R*_1_)/3 Ω; at 2 mA thermometer current it is 4(*R*_2_ − *R*_1_)/3 Ω. The *R*_0_ corresponds to the PRT resistance with no self heating. For temperature measurements of the highest accuracy values of *R*_0_ should be used. (The PRT must have also been calibrated for “zero current” measurements.)

The total self heating of the PRT is described also in terms of the change in the observed resistance caused by the change in the Joule heating or the change in the observed resistance caused by the change in the square of the electric current in the PRT. In this paper this latter coefficient of total self heating will be referred to as the total *self-heating effect.* Referring to eq (10), the total self-heating effect is given by $\left(R_{2}-R_{1}\right)/\left(i_{2}^{2}-i_{1}^{2}\right)$. At the NBS the total self-heating effect is expressed as Ω/(mA)^2^. As given in eq (10) the product of this coefficient and the square of the current being used gives the total self-heating of the PRT.

**Figure 5:**
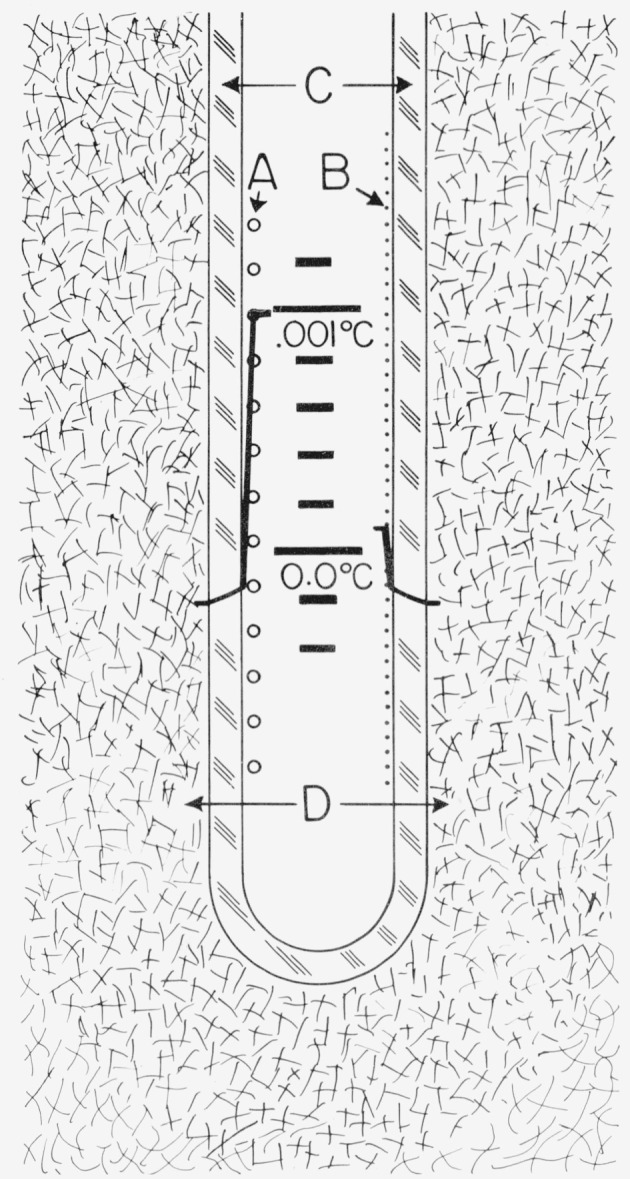
Platinum resistance thermometers at 33 cm immersion in an ice bath. Temperature profile from the midpoint of thermometer coil out to the ice bath with 1 milliampere current.
Platinum coils of coiled filament thermometer; only coils of one side are indicated.Platinum coils of single layer helix thermometer; only turns of one side are indicated.Borosilicate glass thermometer envelope.Finely divided ice and water.

**Figure 6:**
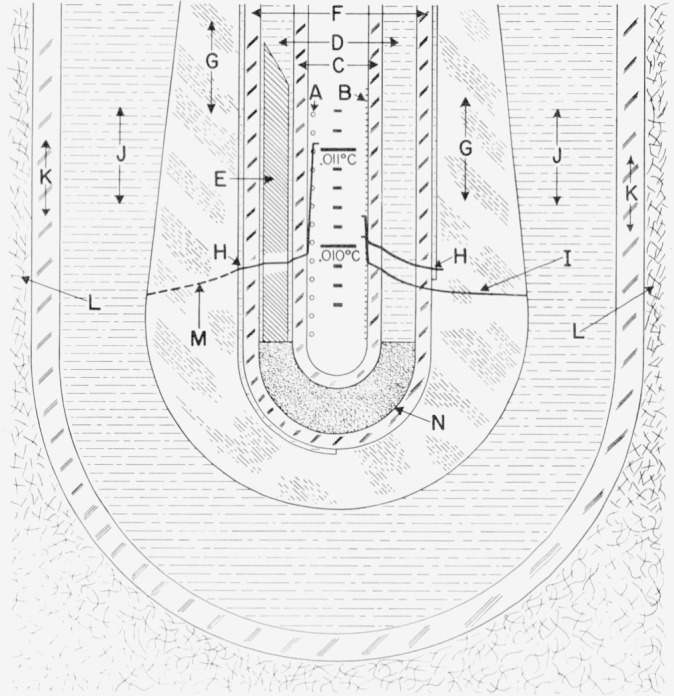
Platinum resistance thermometers at 33 cm immersion in a water triple point cell. Temperature profile from the midpoint of thermometer coil out to the ice-water interface with 1 milliampere current.
Platinum coils of coiled filament thermometer; only coils of one side are indicated.Platinum coils of single layer helix thermometer; only turns of one side are indicated.Borosilicate glass thermometer envelope.Water from ice bath.Aluminum bushing (length not to scale).Borosilicate glass thermometer well.Ice mantle.Inner melt.No inner melting; temperature profile relative to the temperature of outer ice-water interface.Water in cell.Cell well (borosilicate glass).Outside ice-water bath.Temperature profile of the ice mantle.Polyurethane sponge.

A typical steady-state profile of the radial temperature distribution that is expected to result from a current of 1 mA flowing in a 25-Ω PRT (a PRT with the *R*(0 °C) of about 25.5 Ω) immersed in an ice bath and in a TP cell (with and without an aluminum bushing) is shown in figures 5 and 6, respectively. [The profile was calculated from *R*_0_ and *R*_1_ (see eq 11) and the thermal conductivities and thickness of the various parts.] When the outer surface of the protective sheath of the PRT is in direct contact with the surrounding heat sink, the condition closely represented by the PRT immersed in an ice bath (fig. 5) or in a stirred liquid bath, the total self-heating is very nearly the internal self-heating of the PRT. There is no temperature drop beyond the outer surface of the PRT immersed in the ice bath. However, in the TP cell (fig. 6) the PRT is not in direct contact with the heat sink and, therefore, there is an additional temperature drop from the outer surface of the PRT to the water-ice interface (heat sink) of the inner melt where the temperature is very nearly that of the TP except, as previously mentioned in section 2.1, for the effect of the hydrostatic head of water. The external self heating of the PRT in the TP cell is the difference between the total self heating of the PRT in the TP cell and in the ice bath. At the NBS, unless requested otherwise, the PRT’s are calibrated with a continuous current of 1 mA. The calibration (or the observed resistance) is related to the temperature of the outer surface of the protective sheath of the PRT adjacent to the resistor. In the TP cell this temperature corresponds to the temperature of the water-ice interface at the level of immersion of the PRT adjusted for the external self heating of the PRT. In the calibrations at the other fixed points (tin, zinc, and oxygen) the observed resistances of the PRT at a continuous current of 1 mA are also correlated with the temperature of the outer surface of the PRT, i.e., the temperature of the fixed point is adjusted for the external self heating of the PRT in the fixed-point apparatus. Therefore, to employ the PRT calibrations obtained at the NBS directly in temperature measurements, a continuous current of 1 mA must be used and the PRT must be in good thermal contact with the system of which the temperature is being determined so that the external self heating is negligible. Otherwise, a correction must be made for the external self heating. Wherever the highest accuracy is desired or the external self heating is difficult to determine, the two-current method (described earlier) should be used to obtain the zero current value of the PRT resistance. Again the calibration of the PRT must have been determined for zero thermometer current.

## 5. Thermometer Immersion

A PRT is sufficiently immersed in the bath when there is no net heat flow between the sensor and its environment (i.e., the environment external to the bath) through the thermometer leads and the sheath that extends from the region of the sensor. The adequacy of thermometer immersion can be readily checked by measurements at two or three different depths of immersion, allowing for the difference in temperature of the bath at different depths. If the temperatures at different depths do not agree within the desired limits, then the immersion of the PRT is inadequate.

**Figure 7:**
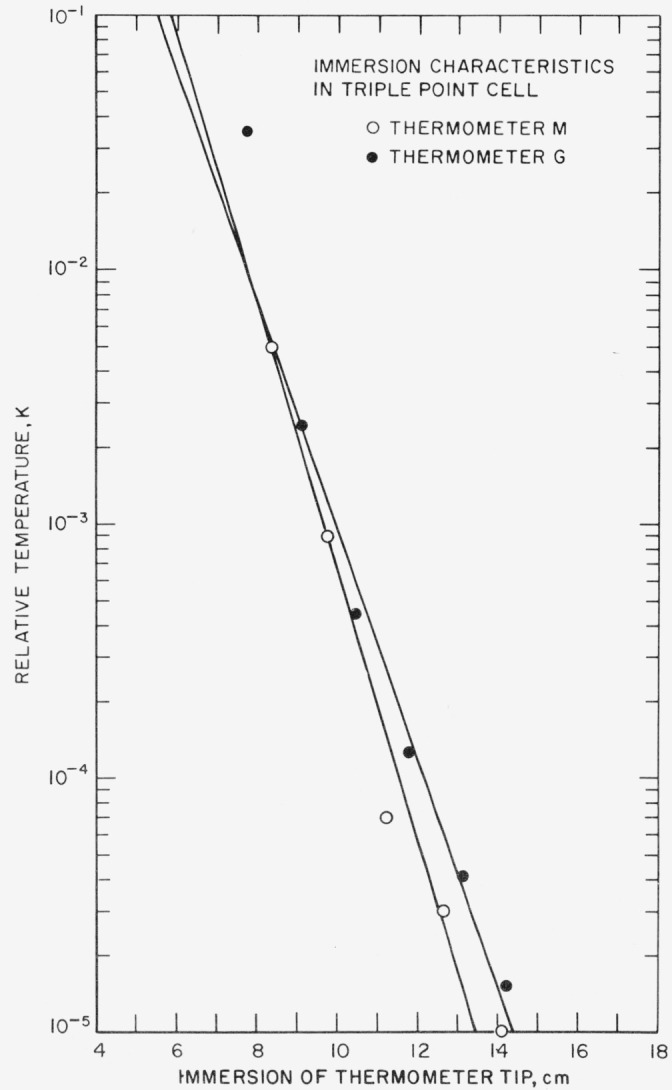
Immersion characteristics in an ice bath of two SPRT’s with different sheath materials and internal construction. The data show the nearly linear relationship between the logarithm of the relative temperature and the depth of thermometer immersion.

**Figure 8:**
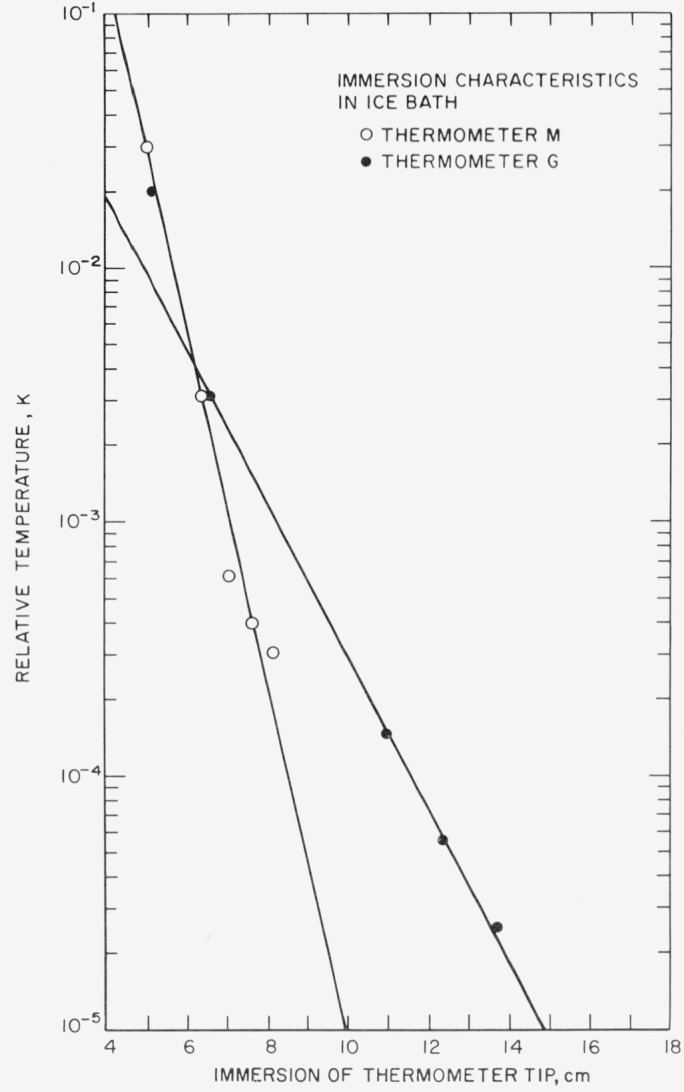
Immersion characteristics in the triple point of water cell of two SPRT’s with different sheath materials and internal construction. The plot shows the relationship between the logarithm of relative temperature and the depth of thermometer immersion. Comparison with figure 7 shows that the immersion characteristics of the thermometers are poorer in the triple-point cell than those in the ice bath. This degradation in the immersion characteristics is caused by the higher resistance to radial heat flow in the triple-point cell.

The required immersion (or the heat conduction along the PRT stem) can be reduced by choosing materials of poor thermal conductivity for the leads from the PRT sensor and for the PRT sheath and by keeping their cross section as small as practicable. The thermometer sensor and its leads (gold or platinum for standards- type PRT) should make as good a thermal contact as possible with the sheath. The convection in the thermometer exchange gas should be localized (e.g., by means of baffles). In use, as previously emphasized, the thermal contact between the PRT sheath and the system of interest should be made as good as practicable. Figure 7 illustrates the case where the PRT sheath is in good thermal contact with the bath (ice-bath heat sink); the figure shows on a logarithmic scale the difference between the ice-bath temperature and the indicated temperature as a function of immersion for two PRT’s of different sheath materials and internal construction. (The measurements shown in figs. 7 and 8 are zero-current values.) For thermometer G, the temperature difference between the thermometer and the bath is reduced by a factor of 10 for each 3.3 cm increase in immersion; for thermometer M, the temperature difference is reduced by a factor of 10 for each 1.4 cm increase in immersion. The relation may be approximated by

$$D=N{\;\log}_{10}\left(\theta /\delta T\right),$$ (12)

where δ*T* is the desired limit of immersion error, *θ* is the difference between the ambient temperature and the bath temperature, *N* is the change in the immersion that is required to change the temperature difference relative to the ice point by a factor of 10, and *D* is the immersion that is necessary to limit the immersion error to δ*T.* Thus, in the case of the thermometers shown in figure 7 with the ambient temperature of 25 °C, in order for the immersion error to be 0.025 mK or less, the thermometer must be immersed enough so that the temperature difference of 25 °C is reduced by six orders of magnitude; i.e., thermometer G must be immersed 19.8 cm and thermometer M must be immersed 8.4 cm in the ice bath.

Figure 8 shows the immersion characteristics of the same thermometers G and M in a TP cell where the thermal contact between the PRT sheath and the heat sink is slightly inferior than in the ice bath. The immersion characteristics of both PRT’s are different from those in the ice bath, particularly for thermometer M where the total immersion must now be about 13.5 cm instead of 10 cm in order for the temperature difference between the observed value and TP temperature to be 0.01 mK. For thermometer G the total immersion required remains nearly the same. The reduction in the thermal contact (see fig. 6) is caused principally by the thermal resistance of the layer of water in the thermometer well and of the glass wall of the well. The relation between the radial and vertical heat flow conditions for thermometer G was not affected as much as in thermometer M by immersion in the TP cell. The radial heat conduction within thermometer G is relatively low; hence, the change in thermal contact from that in the ice bath to that in the TP cell does not result in a large change in the immersion characteristics of the PRT. However, in the case of thermometer M with relatively good radial heat conduction the lower radial heat conduction in the TP cell caused the immersion characteristics of thermometer M to become poorer.

The adequacy of immersion of the PRT in a TP cell can be best checked by measurements at three depths of immersion, e.g., at the bottom, 3 cm up, and 6 cm up from the bottom. If the immersion is adequate at the bottom and at 3 and 6 cm up from the bottom, the PRT readings should track the effect of the hydrostatic head of water in the TP cell, i.e., the readings at 3 and 6 cm up from the bottom should be about 21 and 42 *μ*K, respectively, higher than that at the bottom. The relatively small effect of the hydrostatic head in the TP cell may be obscured by the variations in the total self heating (see sec. 4 on self-heating effects) because of the variations in the thermal contact of the PRT in the TP cell when the vertical location of the PRT is adjusted. Hence, it is essential to carry out two-current measurements and obtain the zero-current resistance readings, which eliminates the effects of self heating, to test the effect of hydrostatic head of water in the TP cell. (At the NBS, as mentioned previously, the PRT’s are immersed 29 or 33 cm depending upon the depth of the thermometer well of the TP cell.)

The adequacy of immersion in the tin-point cell or the zinc-point cell can be checked by the same procedure as employed with the TP cell. In the case of the tin-point cell^12^ the reading at 3 and 6 cm up from the bottom should be about 66 and 132 *μ*K, respectively, lower than that at the bottom and in the case of the zinc-point cell the reading at 3 and 6 cm up from the bottom should be about 81 and 162 *μ*K, respectively, lower than that at the bottom, if the immersion of the PRT is adequate at the three levels in the cells. (At the NBS the mid-points of the PRT sensors are immersed about 18 cm below the liquid surface of the tin and zinc cells. Moreover, to improve the “immersion characteristics” the PRT’s are tempered by the graphite heat shunts located above the graphite crucible containing the metal sample; see fig. 2.)

In the oxygen point apparatus, the copper block ((P), fig. 4) is very nearly isothermal. The adequacy of immersion can be checked by reducing the immersion of the PRT in the copper block. If the “combined immersion” (the tempering through the liquid nitrogen and at the shields and the immersion in the copper block) is adequate the readings at two levels of immersion in the copper block should be essentially the same.

## 6. PRT Calibration Procedure

Unless specifically instructed otherwise by the owner of the PRT, all long-stem type PRT’s that are received for calibration at the NBS are first annealed for about 4 h in a tube furnace held at 480 °C. After annealing, the PRT’s are removed from the furnace and allowed to cool in air at the ambient conditions. (Henceforth, unless indicated otherwise, the long-stem type PRT that meets the IPTS-68 specifications [2] will be referred to as SPRT. The platinum resistance thermometers in general will continue to be referred to as PRT.) The calibration measurements are then obtained at the fixed points in the following sequence: TP, zinc point, TP, tin point, and TP. As mentioned earlier, the SPRT’s are usually calibrated in groups of six. The six SPRT’s are first successively calibrated at the TP, then at the zinc point, and so forth. The calibration at the oxygen point is carried out by the comparison method, employing reference SPRT’s which, as mentioned earlier, are periodically checked to be consistent with the oxygen point maintained on the capsule type reference PRT’s that are used to maintain the NBS–IPTS-68 scale in the region from 13.81 K to 90.188 K [4]. After the oxygen point calibration, another measurement is made on the SPRT at the TP. (Henceforth, for convenience, the four SPRT resistance readings at the TP will be referred to as *R*(TP)_I_ *R*(TP)_Z_, *R*(TP)_T_, and *R*(TP)_O_, respectively, and, when necessary, the values of *R*(0 °C) obtained from these values of *R*(TP) will be given the corresponding subscripts.) Whenever the change in the resistance of the SPRT at the TP is greater than what corresponds to about 0.75 mK during the course of calibration the complete calibration process, including the initial annealing at 480°C, is repeated. In some thermometer designs the changes in the *R*(TP) readings have been found to be on the average greater than in other designs. For these SPRT’s a change in resistance at the TP corresponding to 1 mK is allowed.

Above 0°C, the constants *A* and *B* of eq (6) are obtained by simultaneous solution from the resistance measurements at the TP, the zinc point, and the tin point. The values of the resistance ratio *W*(*T*) corresponding to those at the zinc point and the tin point are calculated using the values of *R*(TP) obtained after the measurements at the metal fixed points; i.e., *W*(Zn)*=R*(Zn)/*R*(0°C)*_z_* and *W*(Sn)*=R*(Sn)/*R*(0°C)*_T_.* (For the calculation of *R*(0°C) from *R*(TP), see section 2.1.) Employing eqs (5) and (6) and the constants *A* and *B*, tables of *W*(*t*) are calculated at integral values of *t* from 0°C to 631 °C for SPRT’s with a fused quartz sheath or from 0 °C to 500 °C for SPRT’s with borosilicate glass or stainless steel sheaths. If a SPRT is also calibrated at the oxygen point, the constants *A*_4_ and *C*_4_ of the deviation function (see eq (4)):

$$\Delta W\left(t\right)=A_{4}t+C_{4}t^{3}\left(t-100{}^{\circ}C\right)$$ (13)

are calculated from the relations:

$$A_{4}=\alpha -\alpha *$$ (14)

and

$$C_{4}=\left[\Delta W\left(t_{0_{2}}\right)-A_{4}t_{0_{2}}\right]/t_{0_{2}}^{3}\left(t_{0_{2}}-100{}^{\circ}C\right),$$ (15)

where α*(= 3.9259668 × 10^−3^ °C^−1^) is the *α* that corresponds to the IPTS-68 reference function [2]. From eq (2)

$$\Delta W\left(t_{0_{2}}\right)=W\left(t_{0_{2}}\right)-W*\left(t_{0_{2}}\right),$$ (16)

where

$$W\left(t_{0_{2}}\right)=R\left(t_{0_{2}}\right)/R{\left(0{}^{\circ}C\right)}_{0}.$$ (17)

Tables of *W(t*) are calculated at integral values of *t* from −183 °C to 0°C using eq (13), the constants *A*_4_ and *C*_4_, and the reference function (eq (3)). The above relations for the SPRT are extrapolated to −200 °C for those laboratories that employ the normal boiling point of nitrogen instead of that of oxygen in calibrating other PRT’s. (In such tables the values of *t* below −183 °C are fictitious and serve primarily as artifices for calibrating other PRT’s for use above −183 °C.)

When the SPRT is calibrated only at the TP, tin point, and zinc point, the tables of *W*(*t*) start from −50 °C. The extrapolation of *W*(*t*) downward to −50 °C is obtained by assuming a value for *C*_4_(≈ 1.7 × 10^−14^ °C^−4^) in the deviation function (eq (13)). The variation in *C*_4_ of SPRT’s is not more than ± 1 × 10^−14^ °C^−4^. By differentiating eq (13) and substituting −50 °C for *t* there is obtained:

$$d\Delta W=1.875\times 10^{7}dC_{4}.$$ (19)

Since *dt/d*Δ*W* at −50 °C is about 247 °C, the uncertainty in the tabulated value of *W*(−50 °C), because of the assumed value of *C*_4_, corresponds to 0.05 mK, which is negligible.

During the course of calibration large changes in the SPRT resistance can arise from a number of sources. If the SPRT is insufficiently annealed prior to the measurements at the zinc point, the *R*(TP)_Z_ reading can be smaller than the *R*(*TP*)_I_ reading because of some annealing that could occur at the zinc point. Also, if the SPRT were “bumped” against an object during or after removal from the annealing furnace but prior to the *R*(TP)_I_ reading, the *R*(TP)_Z_ reading would be expected to be smaller than the *R*(*TP*)_I_ reading on account of the extra strain resistance that was introduced by the bumping of the SPRT but was later partially removed by annealing at the zinc point. On the other hand, if the SPRT were bumped immediately after the *R*(*TP*)_I_ reading, all of the extra strain resistance introduced by the bumping is most likely not removed by annealing at the zinc point; therefore, the *R*(TP)_Z_ reading could then be expected to be slightly higher than the *R*(*TP*)_I_ reading. If any large strains are introduced into the SPRT just prior to the *R*(TP)_Z_ reading, the *R*(TP)_Z_ reading should be larger than the *R* (TP)_I_ reading. If any strong bumping occurs after the *R* (TP)_Z_ reading, the *R* (TP)_T_ and the *R*(TP)_O_ readings should reflect the effect. If the platinum wire were wound too tightly around the coil form, strains can be introduced when the SPRT is cooled to the oxygen point and the *R*(TP)_O_ reading could be relatively higher than the other *R*(TP) readings. Any changes in the *R* (TP) readings smaller than what correspond to 0.75 mK are considered to arise from the general instability of the SPRT and the limit of precision of routine calibration measurements. Obviously, errors of observations, including errors of recording of data, will be superimposed upon the final data.

Berry [17] recently reported that with PRT’s containing oxygen^13^ in the protective sheath the *R*(TP) readings, following the annealing at 450 °C, increased up to 3 or 4 ppm when exposed to temperatures of 200 to 250 °C over a period of seven days. Depending upon the PRT an increase of 1 to 3 ppm is shown to occur within the first hour of exposure to 200 to 250 °C. Accordingly, the *R*(TP)_T_ readings would be expected to be higher than the *R*(TP)_I_ and *R*(TP)_Z_ readings, the increase being dependent upon how long the PRT was in the tin-point cell. It will be shown later that in general the observed *R*(TP)_T_ readings are indeed somewhat higher than the *R*(TP)_I_ and *R*(TP)_Z_ readings in SPRT calibrations. This oxygen-activated thermal effect on the resistance of the PRT seems to impose a limitation on the precision that can be obtained with PRT’s. However, a change of 3 ppm in the *R*(TP) readings corresponds closely to 0.8 mK; such a large increase in the *R*(TP) readings after the resistance readings at the tin point has seldom been observed. It seems that the length of time (approximately 30 min including the time in the preheating furnace) the SPRT is at the tin-point temperature during calibration is not enough to convert the SPRT to a noticeably higher value of the *R*(TP).^14^ Work is planned to investigate further this recently observed oxygen-activated thermal effect.

**Figure 9:**
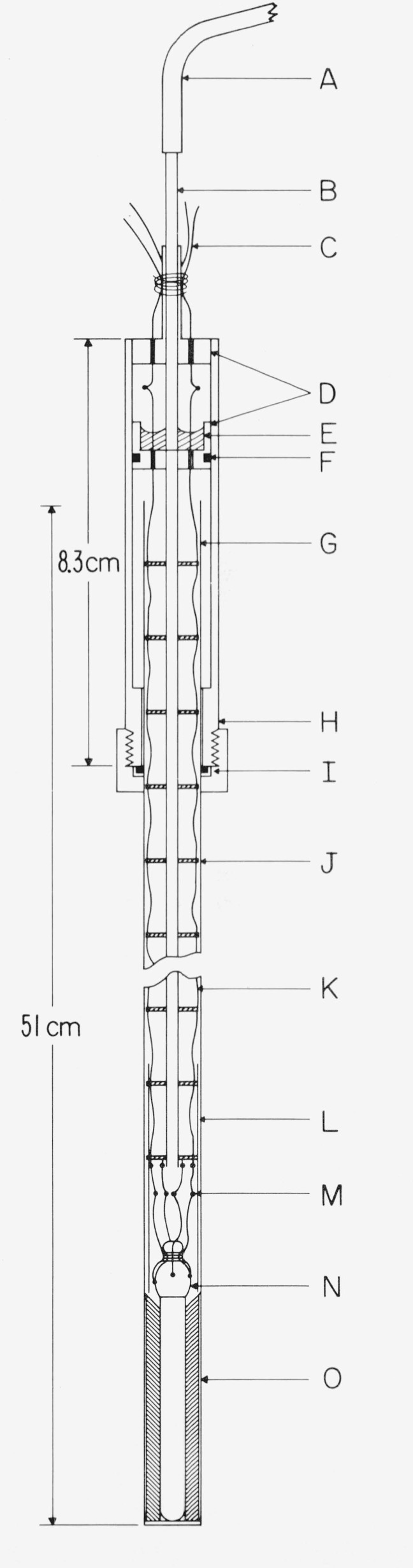
Holder for capsule-type platinum resistance thermometers. Calibration measurements are performed in the holder at the triple point of water, tin point, and the normal boiling point of oxygen. Elastomer tubing to helium gas source.Thin (0.13 mm) wall stainless steel tubing for purging the holder with helium gas before the vacuum tubing connector (I) is sealed.Leads to measurement equipment (Mueller bridge).Sections (brass) of mechanical tie-down, soldered to the stainless purge tube, for guiding and fastening the incoming thermometer leads.Hard wax for holding and sealing the 0.13 mm gold leads that extend down to the thermometer.“O” ring vacuum seal to brass tube (H).Thin wall stainless steel tube (11.1 mm o.d. × 0.11 mm wall) closed at the bottom.Brass tube with lead seal at top and vacuum tubing connector (I) at the bottom.Vacuum tubing connector for sealing the tube (G).Polytetrafluoroethylene plastic lead spacers.Insulated gold leads (4) passing through holes close to the outer diameter of the spacers (J) to attain good tempering. The lead insulation (not shown) is polytetrafluoroethylene tubing cut into a helix and held in tension to eliminate buckling. The four gold leads are welded at the bottom end to short sections of platinum leads.Thin polytetrafluoroethylene sheet rolled into a cylinder to insulate the exposed leads (near the connections to the capsule thermometer) against the stainless steel tubing.Connections between the short sections of platinum leads of the holder and the platinum leads of the capsule thermometer.Capsule thermometer.Aluminum sleeve to fit the thermometer and the stainless steel tube. The sleeve reduces the external self-heating of the thermometer.

Capsule type PRT’s are calibrated as received without annealing. The PRT’s are installed in holders shown schematically in figure 9 for calibration in the TP cell, in the tin-point cell, or in the oxygen-point apparatus. Because of possible damage and the electrical leakage across the metal-glass lead seal at the high temperatures, the capsule type PRT’s are not calibrated at the zinc point as is routinely done with SPRT’s. The capsule type PRT (platinum case with soft-glass lead seal) should not be used at temperatures much higher than about 300 °C. If the PRT is to be used between −183 °C and 300 °C the calibration measurements are made at the fixed points in the following sequence: TP, tin point, TP, oxygen point, and TP. When calibration in the range 13 K to 90 K is requested, the measurement sequence is TP, tin point, and TP, followed by comparison calibration between 13 K and 90 K (see ref. [4] for details of calibration in this range). If the PRT is to be used between −50 °C and 300 °C, the calibration sequence is TP, tin point, and TP.

The tables of *W*(*t*) at integral values of temperature between 0°C and 300 °C are calculated for capsule type thermometers from the calibration measurements at the TP and the tin point and an average value for the coefficient *B* (approximately 5.8755 × 10^−7^ °C^−2^) based on values of about 200 SPRT’s. The range of values of *B* of SPRT’s is about 5 × 10^−10^ °C^−2^. From eq (6)

$$\frac{dW}{dB}=\frac{dA}{dB}t^{\prime }+{t^{\prime }}^{2}.$$ (20)

The coefficient *A* is obtained from the measurements at the TP and the tin point and from the average value of *B.* Assuming that there is no measurement error at the tin point,

$$\frac{dA}{dB}=-231.9292{}^{\circ}C;$$ (21)

then,

$$\frac{dW}{dB}=-231.9292\;t^{\prime }+{t^{\prime }}^{2}.$$ (22)

At *t′* = 300 °C, an uncertainty of ± 2.5 × 10^−10^ °C^−2^ in *B* corresponds to an uncertainty in the value of temperature of about ±1.4 mK. (A survey of values of *B* obtained on SPRT’s received recently shows the average to be closer to −5.8768 × 10^−7^ °C^−2^ and the range to be about 4 × 10^−10^ °C^−2^.) The tables of *W*(*t*) between −183 and 0°C are calculated for capsule type PRT’s by procedures similar to those described earlier for SPRT’s.

## 7. Results of the NBS calibration of PRT’s

### 7.1 R(0 °C) of PRT’s

**Figure 10:**
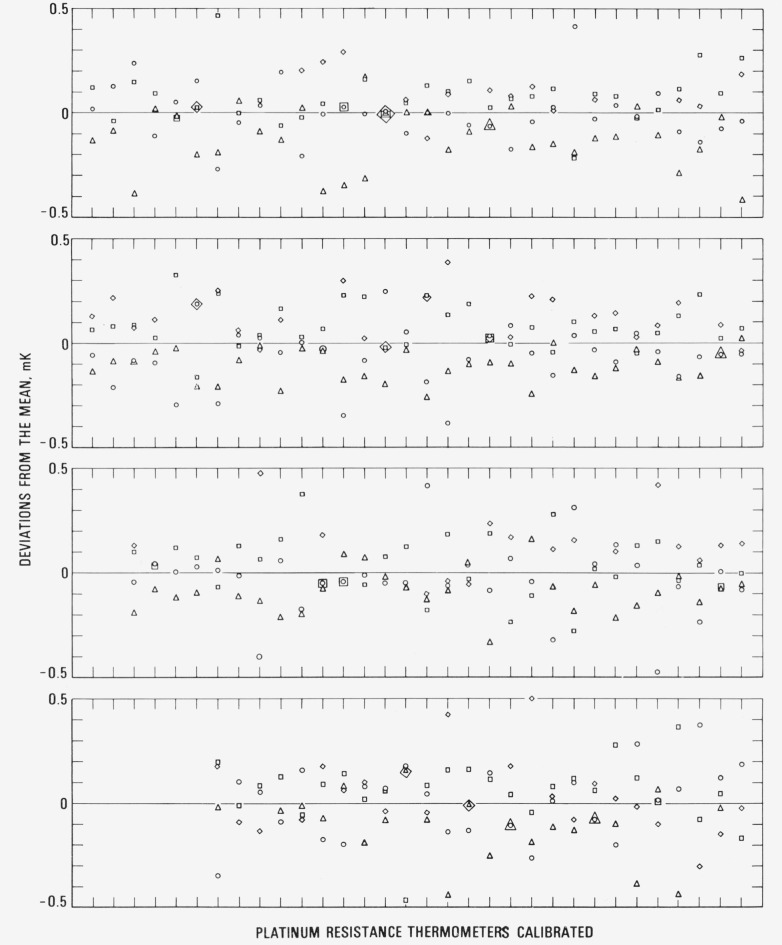
Deviations (in terms of the corresponding values of temperature) from the average value of *R*(0 °C) for each PRT calculated from the observed values of *R*(TP) for PRT’s calibrated between June 1972 and July 1973.

**Figure 11:**
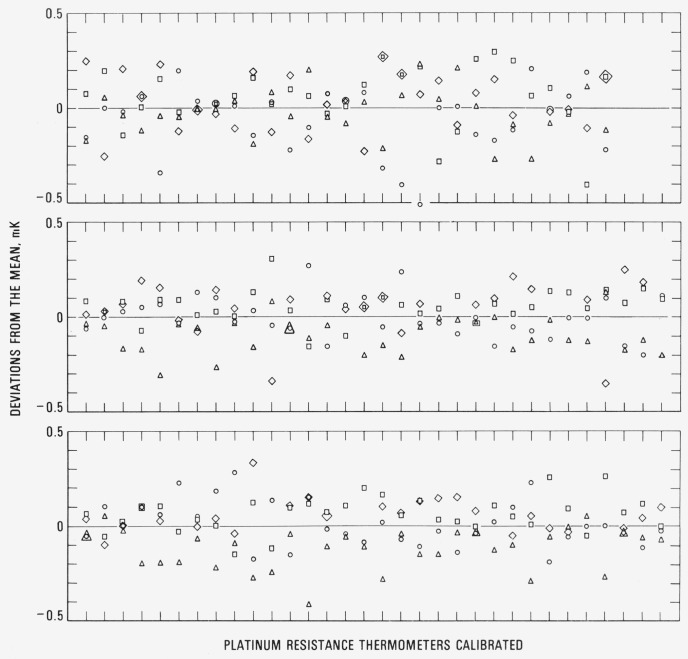
Deviations (in terms of the corresponding values of temperature) from the average value of *R*2(0 °C) for each PRT calculated from the observed values of *R*(TP) for PRT’s calibrated between August 1973 and July 1974.

As indicated earlier, the PRT’s are calibrated after annealing by measuring the thermometer resistances at the fixed points in the following sequence: TP, zinc point, TP, tin point, TP, oxygen point, and TP. Figures 10 and 11 show the variation of the values of *R*(0 °C) for each thermometer about its mean for PRT’s that were received for calibration during a two-year period (June 1972 through July 1974). The symbols ○, △, □, and ◊ correspond to *R*(*0*°C)_I_ *R*(0°C)_Z_, *R*(0 °C)_T_ and *R*(0°C)_O_, respectively, that were calculated from the corresponding values of *R*(*TP*)’s. For most SPRT’s four measurements at the TP are shown. For some SPRT’s the measurements at the oxygen point were not requested; therefore, the *R* (TP)_O_ measurements were not obtained. For capsule type PRT’s, data are shown only when the calibration was requested for the range −183 °C to 300 °C; therefore, the plots correspond to *R*(TP)_I_ *R*(TP)_T_, and *R*(TP)_O_. The standard deviations of the observed *R* (TP)’s for the PRT’s are on the average ±0.15 mK. (As previously mentioned, whenever the range of the observed *R*(TP)’s for the PRT corresponds to 0.75 mK or greater in the initial calibration the PRT is recalibrated; the rejected measurements are not shown in the plot. Also, as previously mentioned, a range of 1 mK in the observed values of *R*(TP)’s is accepted as not atypical for certain designs of PRT’s.) An error in *R*(TP) of ±0.15 mK corresponds to an error in the value of *W* at the tin point of ± 0.30 mK and at the zinc point of ±0.44 mK, assuming that *R*(Sn) and *R*(Zn), respectively, have been determined without error.

The relative values of *R*(TP)’s show certain tendencies; e.g., *R*(TP)_Z_ is more often lower than *R*(TP)_I_, approximately twice as often lower than higher.^15^ However, *R*(TP)_T_ is more often higher than both *R* (TP)_I_ and *R* (TP)_Z_, almost twice as often higher than lower. *R*(TP)_O_ is more often higher than *R*(TP)_T_, approximately $1\frac{1}{3}$ as often higher than lower. These observations suggest that either in cooling from the annealing temperature of 480°C some strains are quenched in, which are removed when heated at the zinc point, or else that the additional heating at the zinc point, following the heating at 480 °C for 4 h, caused more annealing of the PRT. However, the latter seems more consistent since the work of Berry [18] suggests that in cooling rapidly from 500 °C only about 0.1 ppm of *R*(0°C) of strain resistance (a change that corresponds to 0.02 mK) can be quenched in. The observation that values of *R*(TP)_T_ were twice as often higher than those of *R* (TP)_I_ and *R* (TP)_Z_ seem to support the work of Berry [17] that when an annealed SPRT is exposed to temperatures of 200 to 250 °C the *R* (TP) increases 3 or 4 ppm over a period of seven days. Since *R*(TP)_O_ is $1\frac{1}{3}$ times as often higher than it is lower than *R*(TP)_T_, some SPRT’s could have been slightly strained between these two measurements.

### 7.2 Check PRT’s

As part of the calibration process at the NBS, separate check SPRT’s are employed to monitor the constancy of the temperature of each of the fixed points. In the cases of the freezing-point cells of tin and zinc, although the metals employed in the cells are of high purity, the equilibrium temperature of the liquid-solid phases is somewhat dependent upon the relative amounts of the two phases. Usually the SPRT’s are calibrated during the period in which the first 50 percent of the metal is being frozen. Of a group of SPRT’s to be calibrated, the first and the last measurements in each freeze are obtained with the check SPRT. If the second reading on the check SPRT is lower than the first by what corresponds to 0.5 mK some of the SPRT’s being calibrated, based on the time of the measurements that were obtained, are recalibrated employing a new freeze with the cell. The first and the last measurements on the new freeze are again obtained with the check SPRT. The comparison of the results of the repeat calibration with those of previous freeze will show whether SPRT’s calibrated earlier in the sequence with the previous freeze should also be recalibrated. The measurements with the check SPRT of every new freeze are compared with those of the previous freezes. The check SPRT’s employed with the zinc and tin point cells are usually about 1.5 and 2 h at their respective fixed points during each freeze. The check SPRT for the tin point cell is near the tin point longer because of the preparation and manipulation that are followed for initiating the freeze by withdrawing the cell from the furnace well (see sec. 2.3).

For the oxygen point comparison calibration (see sec. 2.4) a second reference standard SPRT is used as the check SPRT to monitor the consistency of the “working” reference standard SPRT. The value of *W*(0_2_) obtained for the check SPRT is compared with those of previous calibrations.

With each of the readings of the check SPRT’s at the fixed points there are also obtained readings at the TP. Thus, there is collected a history of measurements with check SPRT’s and fixed points.

**Figure 12:**
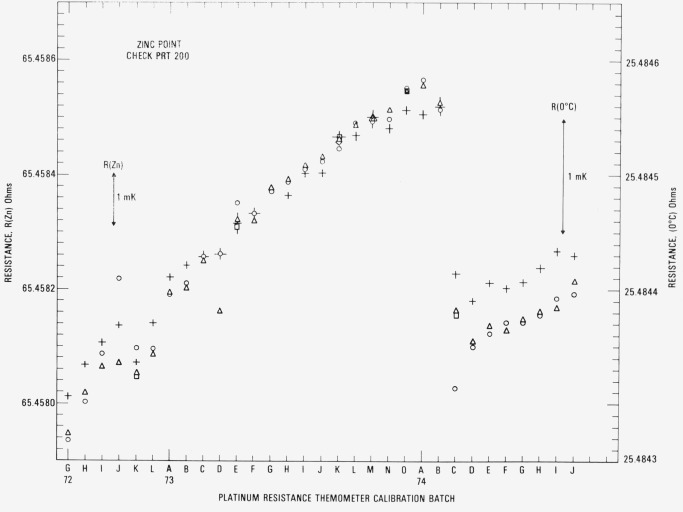
Observed values of resistances for the check SPRT 200 at the zinc freezing point and at the TP (converted to *R*(0 °C)). The sequence of zinc-point observations is represented by the symbols in the order ○, △, □, and ◊; the *R*(0 °C) values are represented by the symbol +.

**Figure 13:**
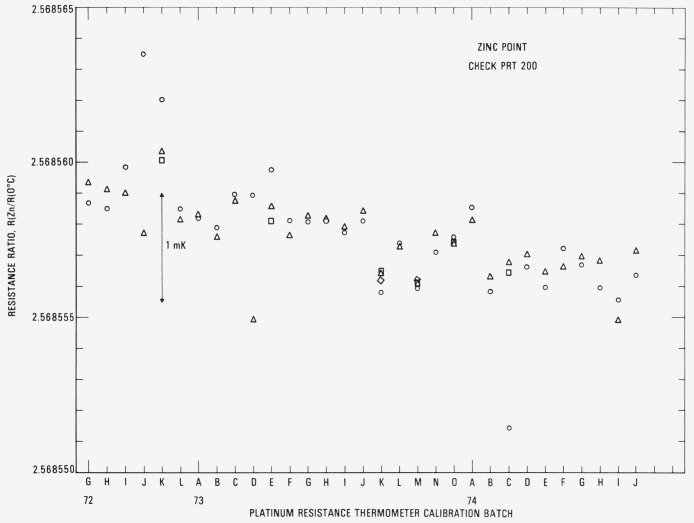
Values of *W* for the check SPRT 200 from observations of the resistances at the zinc freezing boint and at the TP. The sequence of zinc-point observations is represented by the symbols in the order ○, △, □, and ◊.

Figure 12 shows the observed *R*(Zn) and *R*(0°C) for the check SPRT (designated 200) used with the zinc-point cell. The symbols ○ and A indicate in most cases the *R*(Zn) readings at the beginning and end, respectively, of a group of SPRT’s that were calibrated. Occasionally additional *R*(Zn) readings have been obtained, the sequence being ○, △, □, and ◊. When the first reading ○ is suspected and the second reading △ is obtained, the symbols △ and □ indicate the beginning and end readings, respectively, of a group of SPRT’s that were calibrated. The fourth reading, indicated by the symbol ◊, is a check, if considered necessary, on the previous reading indicated by □. In calibration batches 72J and 73D the difference in the beginning and end readings of the check SPRT are shown to be excessive. Some of the test SPRT’s were recalibrated in the next batch. The symbol + indicates the *R*(0°C) readings obtained after the *R*(Zn) readings. There is shown a definite increase in the values of *R*(Zn) and *R*(0°C) associated with each freeze. (Because of possible strains that are introduced, the resistance of PRT’s usually increases with use.) The sharp decrease shown at 74C resulted principally from the change in the Mueller bridge resistance unit which was previously based on one standard resistor. The new resistance unit is based on the average of four standard resistors. However, since the Mueller bridge is calibrated to be “linear,” the values of *R*(Zn)/*R*(0 °C) or *W*(Zn) for the check PRT should be independent of the resistance unit (see fig. 13). The downward trend in the *W*(Zn) reflects the change in the SPRT with use. However, the total change over the two-year period corresponds to not more than 1 mK. In the interval from the calibration batch 74C to 74J (approximately one-half year) *W*(Zn) was found to be fairly constant and its range corresponds to about 0.4 mK. The standard deviation of the values of *W*(Zn) was computed after dividing the data into three intervals: 72G to 72K, 72L to 73J, and 73K to 74J. (Between the intervals the values of *W*(Zn) seem to show a large change possibly from slight bumping of the check SPRT.) The pooled standard deviation was found to correspond to ±0.28 mK.

**Figure 14:**
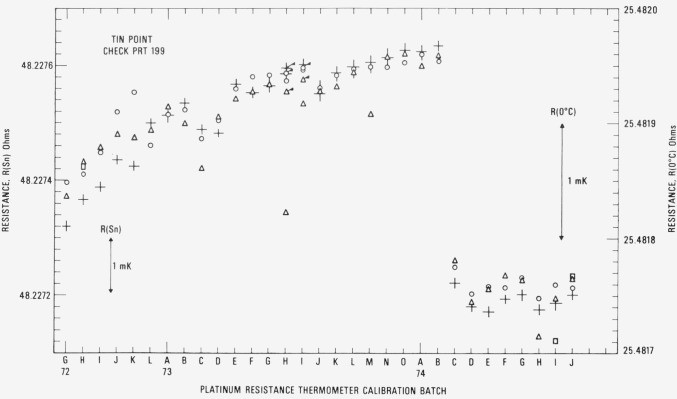
Observed values of resistances for the check SPRT 199 at the tin freezing point and at the TP (converted to *R*(0 °C)). The sequence of tin-point observations is represented by the symbols in the order ○, △, and □; the *R*(0 °C) values are represented by the symbol +.

**Figure 15:**
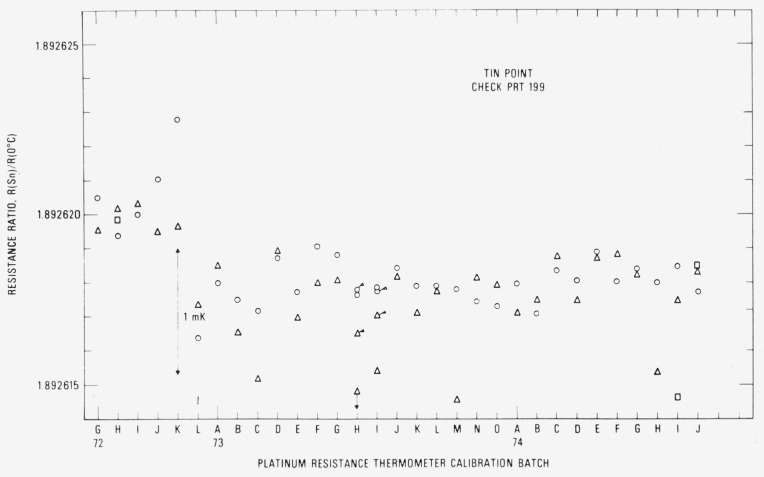
Values of *W* for the check SPRT 199 from observations of the resistance at the tin freezing point and at the TP. The sequence of tin-point observations is represented by the symbols in the order ○, △, and □.

Figure 14 shows the observed *R*(Sn) and *R* (0 °C) for the check SPRT (designated 199) used with the tin-point cell. The symbols have the same significance as those used with the readings obtained with the check SPRT for the zinc-point cell. In two cases 73H and 731, a set of symbols is “flagged.” These indicate readings with a second freeze that was used in the calibration of the group of SPRT’s. Up to calibration batch 74C the trend of the *R*(Sn) and *R*(0 °C) readings is shown to increase, but not as rapidly as in the case with check SPRT 200 for the zinc-point cell. As shown in the measurements with the zinc point check SPRT (see fig. 12), a sudden change in *R* (Sn) and *R* (0 °C) occurs at calibration batch 74C because of the change in the Mueller bridge resistance unit. The reason for the relatively constant readings obtained starting from the calibration batch 74C is at present not known. Perhaps the oxygen activated change in *R*(0 °C) reported by Berry [17] finally reached an equilibrium state for the tin point. (Berry did not report how the thermometer resistances changed at the higher temperatures at which the thermometers were “soaked.” In a more recent work Berry [19] found that the reproducibility of *W* (100 °C) of five SPRT’s corresponded to 0.1 mK as long as the *R* (100 °C) and *R* (0°C) were observed with the platinum of the SPRT in the same oxidation state. The values of *W* (*t*) that are obtained in the NBS calibration procedure discussed in this paper correspond essentially to this requirement. Figure 15 shows the plot of *R* (Sn)/*R* (0 °C) or *W* (Sn) for the check SPRT. The values of *W* (Sn) are significantly more constant than those of *W* (Zn) which indicate that the SPRT is not changed as rapidly when used at the tin point as at the zinc point. (The check SPRT’s 199 and 200 are essentially alike in construction and were obtained at the same time. Another SPRT may show better or worse stability.) From calibration batch 72K to 74J most of the observed values of *W* (Sn) are within ±0.3 mK. The noticeable change in the *W* (Sn) reading after the calibration batch 72K suggests that the check SPRT was “bumped” slightly. (There is a relatively large increase in *R* (0 *°*C) in calibration batch 72L.) The change in the check SPRT indication has no effect on the SPRT’s that were being calibrated. The standard deviation of the values of *W* (Sn) was computed after dividing the data into two intervals: 72G to 72K and 72L to 74J. The pooled standard deviation was found to correspond to ±0.30 mK, which is essentially the same as that (±0.28 mK) found for the values of *W* (Zn) obtained with the check SPRT 200.

**Figure 16:**
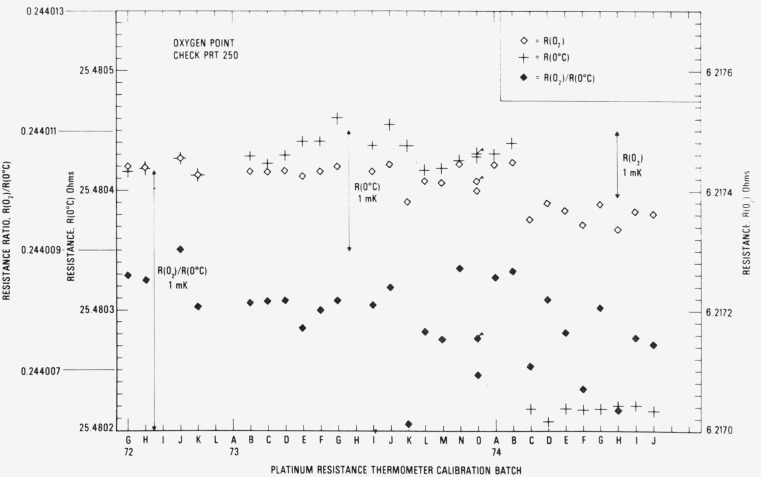
Observed values of resistances for the check SPRT 250 at the oxygen point and at the TP (converted to *R*(0 °C)) and values of *W* calculated from the observations. The oxygen-point observations are represented by the symbol! ◊ and the *R* (0 °C) values are represented by the symbol +. The corresponding values of *W* (O_2_) are represented by the symbol

Figure 16 shows the observed *R* (O_2_) and *R* (0 °C) for the check SPRT (designated 250) used in the intercomparison calibrations at the oxygen point. The symbol ◊ indicates the check measurements at the oxygen point and the symbol + indicates the *R* (0 °C) readings. The “flagged” points for calibration batch 730 indicate that second readings were obtained in the oxygen-point calibration with the group of SPRT’s that were calibrated. (The SPRT’s of the batch were calibrated on two days; therefore, two check SPRT readings were obtained.) The values of *R* (O_2_)/*R* (0 °C) or *W* (O_2_) are also plotted in figure 16. Although there is a small downward trend, the values of *W* (O_2_) are more stable than the values of *W* (Zn) and *W* (Sn) of the check SPRT’s used with the zinc and tin point cells, respectively. Excluding some outlying observations which are shown to deviate relatively more (see calibration batches 73K, 74F, and 74H), the range of *W* (O_2_) corresponds to not more than 0.7 mK. The standard deviation of the values of *W* (O_2_) corresponds to ± 0.16 mK, which is significantly smaller than that found for *W* (Zn) and *W* (Sn) obtained with their respective check SPRT’s.

### 7.3 Calibration of “MAP” Thermometers

As part of the NBS Measurement Assurance Program (MAP) on platinum resistance thermometry a set of three calibrated SPRT’s has been furnished to participating standards laboratories of the nation. On receipt of the SPRT’s the laboratory first determined *R* (TP) at 1 and 2 mA currents and then calibrated the SPRT’s according to its regular laboratory procedure. After completion of the calibration the laboratory determined *R* (TP) at 1 and 2 mA currents just before shipping the SPRT’s back to the NBS. On return of the SPRT’s to the NBS their *R* (TP)’s were first determined at 1 and 2 mA currents; they were then annealed and recalibrated according to the usual retained as the SPRT’s are subjected to conditions of use.

**Figure 17:**
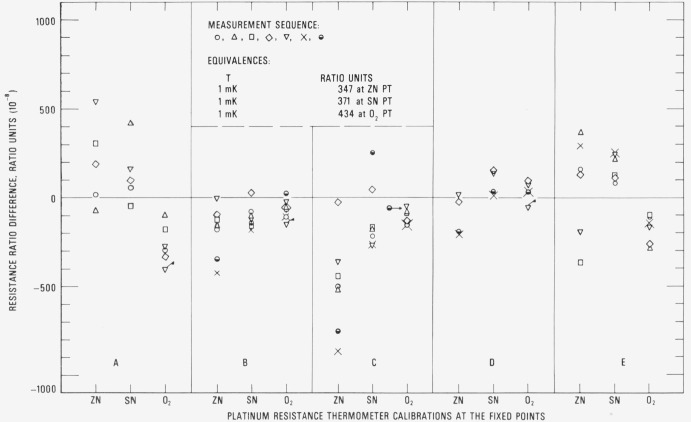
Values of *W* of SPRT’s (relative to the first calibration) obtained at the National Bureau of Standards from a series of calibrations at the TP and at the zinc, tin, and oxygen points. Thermometers A, B, C, and D were employed in the Measurement Assurance Program and thermometer E was calibrated in successive batches.

For the convenience of the comparison of the successive calibrations at the NBS and, at the same time, to show the degree to which the SPRT’s can retain its calibration, the resistance ratios *W* at −183, 232, and 420 °C obtained through the calibration process are compared relative to the first calibration of the series. The results for SPRT’s A, B, C, and D are shown in procedure. The SPRT’s were then shipped to the next participating standards laboratory. The SPRT’s were shipped cushioned in soft plastic foam inside a wooden box. When the SPRT’s were at the various standards laboratories they were subjected to the annealing temperatures (450 °C to 480°C) for 4 h and to the various temperatures (about − 180, 0, 232, and 420 °C) of the fixed points that were employed in the calibration. While at the NBS the SPRT’s were annealed at 480°C for 4 h and then recalibrated. Therefore, in the interval between the calibrations at the NBS the SPRT’s were exposed to temperatures over a wide range, including the higher temperatures where they were “annealed.” The successive calibrations that were obtained at the NBS were examined to determine the reproducibility of the calibration and also to determine how well the calibrations of the SPRT’s are figure 17.^16^ (The first three calibrations of thermometers A, B, and C and the first two calibrations of thermometer D were obtained before shipping them to the standards laboratories that participated in the MAP study.) The calibration sequence after the first calibration is indicated by the symbols ○, △, □, ◊, ∇, ×, and ◒. The flagged points indicate a second measurement using another freeze. There is a tendency for the values of *W* (Zn) to increase with use which was not the observation with the check SPRT 200 (see fig. 13). The calibration is noticeably more scattered at the zinc point than at the tin point or at the oxygen point. The deviations reflect the combined variations in the observations of *R* (TP) and of *R* (Zn), *R* (Sn), or *R* (O_2_), the variations in *R* (TP) being amplified in *W* (Zn) and *W* (Sn) (see sec. 7.1). The deviations should reflect also the effect of handling of the SPRT’s in the various laboratories and the effect of handling during shipment. Table 1 summarizes the estimated standard deviations of the values of *W* (Zn), *W* (Zn), and *W* (O_2_) for thermometers A, B, C, and D and the check SPRT’s. The average of the standard deviations of *W* (Zn) observed for SPRT’s A, B, C, and D is shown to be about twice that observed for the check SPRT 200; only the estimated standard deviation of *W* (Zn) for thermometer D appears to be comparable to that of check SPRT 200.

**Figure 18:**
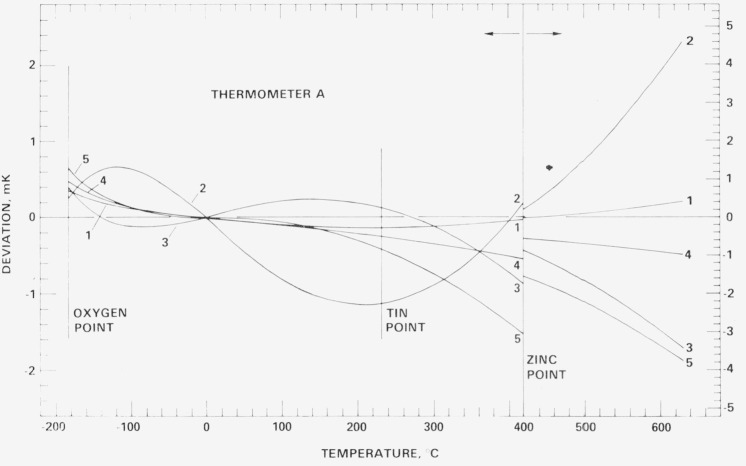
Deviations, relative to the first calibration, of values of *W(t*) (in terms of corresponding values of temperature) of succeeding calibrations of thermometer A.

**Figure 19:**
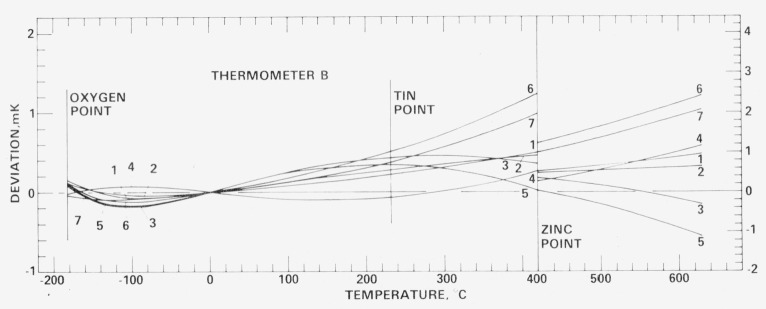
Deviations, relative to the first calibration, of values of *W(t*) (in terms of corresponding values of temperature) of succeeding calibrations of thermometer B.

**Figure 20:**
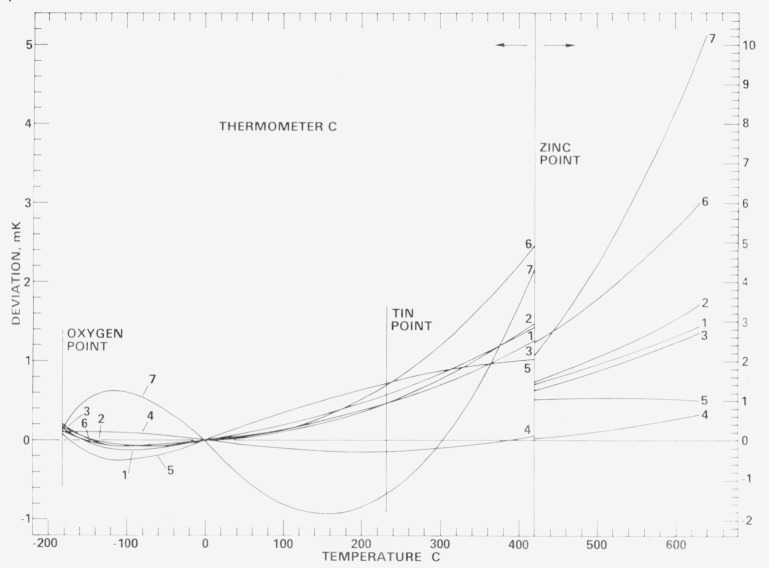
Deviations, relative to the first calibration, of values of *W(t*) (in terms of corresponding values of temperature) of succeeding calibrations of thermometer C.

**Figure 21:**
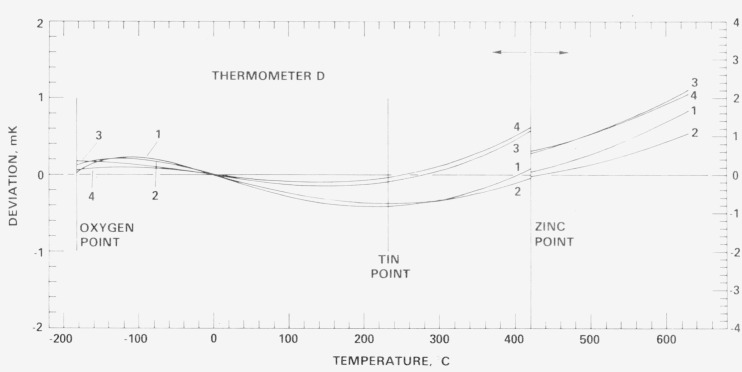
Deviations, relative to the first calibration, of values of *W(t*) (in terms of corresponding values of temperature) of succeeding calibration of thermometer D.

Figures 18, 19, 20, and 21 show for each SPRT how the values of *W* (*t*) (in corresponding values of temperature), based on the various calibrations, deviate over the range −183 °C to 630 °C relative to the first calibration. The figures indicate the levels of uncertainty of temperature measurement that can be achieved, under the various conditions to which the SPRT’s were subjected, relative to the IPTS-68 maintained by the NBS. For example, the degree of repeatability of the measurements at the fixed points indicates the degree of retention of calibration by the SPRT. Since the standard deviations of the values of *W* (Sn) and *W* (O_2_) are comparable and those of *W* (Zn) are about twice those observed with the check SPRT’s, the calibration of the SPRT’s have been retained close to the limit of calibration measurements at the NBS. The user of a SPRT that was calibrated at the NBS can look upon figures 18, 19, 20, and 21 to indicate the level of retention of calibration (after annealing in a similar manner) and repeatability of calibration of a SPRT and, in the analysis of his temperature measurements, should combine the uncertainties shown in the figures with the uncertainties of his measurements to obtain his overall uncertainty relative to the NBS.

### 7.4 Repeated calibrations

As another test of reproducibility of the NBS calibrations at the fixed points, a SPRT was repeatedly calibrated (including the initial annealing) in seven successive batches of calibrations. The sequence of calibration of this “test” SPRT (thermometer E) was varied within each batch of SPRT’s that were to be calibrated for the customers so that the location of calibration on the freezing “plateau” of the zinc- and tin-point cells was varied for the SPRT. The values of *W* that were obtained at the zinc, tin, and oxygen points are compared (relative to the first calibration in batch 74D) in figure 17 along with those calibrations obtained for thermometers A, B, C, and D that were used in the NBS–MAP measurements described earlier. The standard deviations of the measurements are summarized in table 1. The results show that the variations in the calibrations of thermometer E are comparable to those obtained for thermometers A, B, C, and D; therefore, the additional handling and “use” of the NBS–MAP therometers did not seem to have affected the calibration of these SPRT’s to a large extent. (These measurements were on only one thermometer E; other SPRT’s may show better or worse reproducibility.)

**Figure 22:**
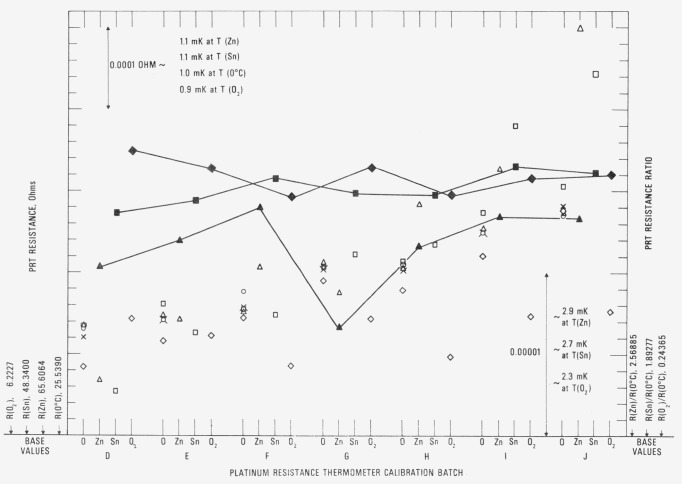
Observed values of resistances at the TP (converted to *R*(0 °C)) and at the zinc, tin, and oxygen points and the values of *W* calculated from the observations for thermometer E calibrated successively in the batches 74D to 74J. The sequence of observations at the TP is represented by the symbols in the order ○, △, □, and ◊. The open symbols △, □, and ◊ represent the observations of resistance at the zinc, tin, and oxygen points respectively; the corresponding filled-in symbols represent the values of *W* at these points.

**Figure 23:**
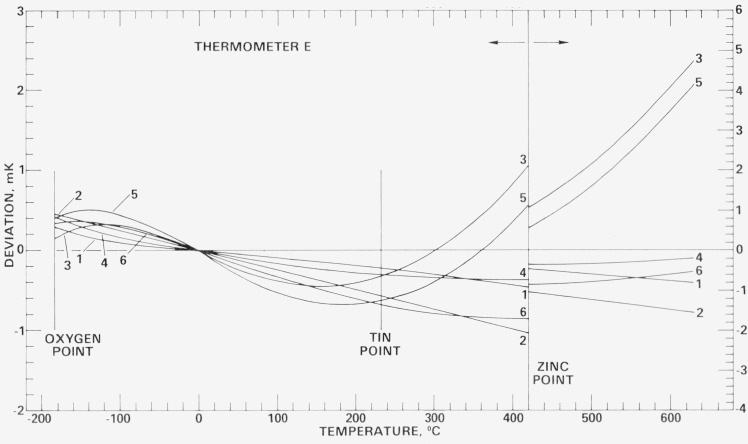
Deviations, relative to the first calibration, of values of *W(t*) (in terms of corresponding values of temperature) of succeeding calibration of thermometer E.

Figure 22 shows more clearly how the observations obtained with thermometer E changed with each calibration. The symbols △, □, and ◊ are associated with the measurements at the zinc, tin, and oxygen points, respectively. The same symbols are used to represent the observations at the TP after the measurements at the above three fixed points. The symbol ○ represents the initial measurements at the TP after annealing the SPRT. The filled in symbols represent the values of *W*(*t*) at the corresponding fixed points. The plot shows that the values of the SPRT resistances tended to increase with each calibration.^17^ However, the values of *W*(*t*) obtained at the fixed points do not show any definite trend. Figure 23 shows how the values of *W*(*t*) (in terms of equivalent values in *t*) vary relative to those of the first calibration. The measurements of the first calibration were slightly outside of those measurements obtained in the later series of calibrations.

## 8. Discussion and Conclusion

The calibration data on 213 PRT’s (mostly SPRT’s) that were received over a two-year period show that the average of the standard deviations of *R* (TP)’s that were observed for each of the thermometers corresponds to ±0.15 mK. This indicates the precision of the NBS calibration measurements and the high reproducibility of the SPRT’s at the TP and the high stability of the SPRT’s even after being subjected to temperatures as high as 420 °C or as low as − 183 °C. The high reproducibility of values of *W* of the check SPRT’s (serial nos. 199, 200, and 250) that are used for the zinc, tin, and oxygen points (pooled standard deviations: ±0.28, ±0.30, and ±0.16 mK, respectively) shows that SPRT’s can be employed in high-precision temperature measurements. [An error in *R* (TP) is amplified (or attenuated) in the value of *W* at these fixed points by 2.6, 1.9, and 0.24 times, respectively.] The high reproducibility also shows that the tin and zinc points and the reference standard SPRT for the oxygen point are highly stable.

Although both the thermometer resistances and resistance ratios were found to change, the resistance ratios were more stable. The data on the check SPRT’s were taken over a two-year period; measurements over a longer period or perhaps other conditions are needed to determine the useful life of a SPRT.^18^ The values of *W* (Zn) of the check SPRT used with the zinc point are decreasing slightly with use. An annealing of this SPRT at a higher temperature (e.g., 480 °C) may reduce this trend. The SPRT’s that were used in the MAP study and in repeated calibrations were annealed at 480 °C prior to each calibration; the values of *W* (Zn) are either randomly scattered or are increasing slightly with use.

The results with the SPRT’s used in the MAP study show that when the thermometers are shipped in a suitable protective container the calibration is retained. (The SPRT’s have been shipped and received from a number of laboratories, some located as far as across the continent.) The results show also that SPRT’s can be employed reliably in precise temperature measurements. When these SPRT’s were heated at 450 °C or 480 °C for 4 h and calibrated at each of the laboratories (5) that participated in the MAP study and were heated again at 480 °C for 4 h at the NBS when the SPRT’s were returned from each of the laboratories, the calibrations did not change by more than ±1 mK.^19^ The variations in the observed values of *W* reflect the imprecision in the calibrations superimposed upon whatever changes that have occurred in the SPRT’s. The largest scatter was at the zinc point, the average of the standard deviations being ±0.6 mK. The average of the standard deviations of the observed values of *W* of the SPRT’s at the tin and oxygen points were ±0.3 mK and ±0.2 mK, respectively. These standard deviation figures for the tin and oxygen point measurements are comparable to those obtained with the check SPRT’s which suggest that the treatments to which the MAP SPRT’s were subjected did not significantly affect the thermometers anymore than if the SPRT’s had remained at the NBS laboratory and been used. On the other hand, the standard deviation figure obtained on the MAP SPRT’s at the zinc point is about twice as large as that obtained with the check SPRT. The values of *W* that were obtained at the three fixed points in the repeated calibrations of a SPRT were similar to those obtained with the MAP SPRT’s. Further work is needed to determine the source of larger imprecision at the zinc point.

The PRT calibration on the IPTS-68 is based on relatively few fixed points. A small error (e.g., corresponding to 1 mK) at any of the fixed points can go undetected and cause large errors above the zinc point (see figures 18, 19, 20, 21, and 23). A fixed point near the upper limit of the PRT scale would be a sensitive means of detecting calibration errors at the tin or the zinc point; or if the PRT is to be used primarily at the higher temperatures, the freezing point of antimony (630.755 °C) or the freezing point of aluminum (660.46 °C) could be included in the calibration [20]. Measurements at the freezing point of cadmium (321.108 °C) could also serve to check the IPTS-68 calibration. Below 0°C the triple point of xenon (−112 °C) could perhaps be developed for checking the calibration. The comparison calibration of test PRT’s in terms of two or three reference standard PRT’s, which are periodically “checked” on the IPTS-68, can yield many calibration points that can be analyzed by a least squares method so that the contribution from an error in any one observation would be negligible. Investigations are in progress to improve the calibration accuracy of PRT’s.

## Figures and Tables

**Table 1:** Estimated standard deviation of values of *W* at the fixed points of some platinum resistance thermometers

SPRT	*W* (*Zn*)		*W* (Sn)		*W* (O_2_)	
	*±W ×*10^−8^	*±t* mK	*±W ×*10^−8^	*±t* mK	*±W ×*10^−8^	*±t* mK
A (6)^a^	230	0.66	165	0.46	142	0.33
B (8)	150	.43	76	.21	55	.13
C (8)	308	.88	184	.51	55	.13
D (5)	109	.31	73	.20	60	.14
Mean	199	.57	124	.33	78	.18
E (7)	261	.75	92	.25	98	.23
Mean^b^	212	.61	118	.32	82	.19
Check						
SPRT^c^	99	.28	110	.30	71	.16

a Numbers in parentheses indicate the number of calibrations.

b Mean of SPRT’s A, B, C, D, and E.

c *W* (Zn) was observed with check SPRT 200, *W* (Sn) with check SPRT 199, and *W* (O_2_) with check SPRT 250.

## Footnotes

1 Figures in brackets indicate the literature references at the end of this paper.

2 The tin point (231.9681 °C) is an alternative to the steam point (100 °C) and the argon triple point (−189.352 °C) is an alternative to the oxygen normal boiling point (−182.962°C).

3 In an experiment where the ice mantles of five cells were intentionally cracked during the preparation of the cells, the TP temperatures observed for the cells did not differ significantly, if at all, from the temperatures that were obtained when the cells were prepared without cracking the mantles. This indicates that the crack healed sufficiently so that it did not cause the water outside the mantle to mix with that in the inner melt or that the water of the TP cell is highly pure.

4 On occasions when a layer of ice formed across the top, the internal pressure that was formed broke the ice layer instead of the glass cell. The water that escaped through the break in the ice immediately became an “ice fountain.”

5 The water level in the cell serves as an indicator of whether an adequate (or an excessive) amount of ice is formed around the thermometer well.

6 Of the five TP cells where the inner and the outer water were intentionally mixed by alternately inverting and uprighting the cells, the TP temperatures that were observed for four of the cells were essentially the same as those before mixing. For the fifth cell the TP temperature after mixing was about 0.04 to 0.05 mK lower than before mixing [5].

7 Visible and near-infrared radiations transmitted through the ice bath could easily cause errors as large as 0.5 mK [6]. The aluminum sleeve can help shield the PRT sensor from the radiation.

8 In 1966 the PRT Calibration Laboratory of the NBS was moved from Washington, D.C. to the new NBS laboratories in Gaithersburg, Md. The zinc point and the tin point were activated instead of the sulfur point and the steam point, respectively.

9 Certain commercial materials and instruments are identified in this paper in order to adequately specify the exprimental procedure. Such identification does not imply recommendation or endorsement by the National Bureau of Standards.

10 1 Torr = 133.322 Pa.

11 More recently the Mueller bridge resistance has been based on the average comparison with four 100-Ω resistors which had been calibrated in terms of the NBS absolute ohm.

12 At the NBS the tin-point cell is used without the inner freeze immediately next to the thermometer well. Because of the liquid-solid interface not being close to the thermometer well the temperature gradient expected from the change in pressure with depth is somewhat obscured; the temperature observed at 3 and 6 cm up from the bottom is very nearly the same as that at the bottom. However, the temperature observed at the bottom is essentially the same as that observed at the bottom with an inner freeze immediately next to the well.

13 Dry air is sealed in the protective sheath of most SPRT’s. Most capsule-type PRT’s have helium gas with a small amount of oxygen sealed in the capsule. Where pure helium gas was initially used, it is possible that the gas would eventually become contaminated with some oxygen.

14 In an earlier investigation on the freezing point of tin [11] the SPRT that was employed was initially annealed at 480 °C. Before the intercomparison measurements of tin-points were started the SPRT was probably used in preliminary experiments at the tin point for a total of about 6 h. The subsequent measurement described in reference [11] shows that *R*(TP) increased by an amount corresponding to about 0.01 to 0.02 mK per 1 h and 40 min exposure at the tin point.

15 Since *R*(TP) was actually observed, reference is made here to *R*(TP). In figs. 10 and 11 the plot is in terms of *R* (0 °C). However, in this discussion and in the plot *R*(TP) and *R* (0 °C) are interchangeable since only their relative values are being examined.

16 After several successive calibrations the relatively older thermometer A was considered to be less stable than thermometers B and C. Thermometer A was subsequently replaced with thermometer D.

17 In these measurements the same Mueller bridge calibration was used. Neither the history of the Mueller bridge nor subsequent calibrations indicate that the drift of the bridge was responsible for the drift shown in fig. 22.

18 A SPRT is usually broken before instabilities resulting from grain growth or other defects cause it to be “retired.”

19 The SPRT’s were “annealed” at 480 °C before they were calibrated. The reproducibility of the measurements at the fixed points may have been less if the SPRT’s had not been annealed.
